# Phenotypic Complexity, Measurement Bias, and Poor Phenotypic Resolution Contribute to the Missing Heritability Problem in Genetic Association Studies

**DOI:** 10.1371/journal.pone.0013929

**Published:** 2010-11-10

**Authors:** Sophie van der Sluis, Matthijs Verhage, Danielle Posthuma, Conor V. Dolan

**Affiliations:** 1 Functional Genomics Section, Department of Clinical Genetics, Center for Neurogenomics and Cognitive Research (CNCR), VU University and VU University Medical Center, Amsterdam, The Netherlands; 2 Medical Genomics Section, Department of Clinical Genetics, Center for Neurogenomics and Cognitive Research (CNCR), VU University and VU University Medical Center, Amsterdam, The Netherlands; 3 Department of Psychology, Faculty of Social and Behavioural Sciences (FMG), University of Amsterdam, Amsterdam, The Netherlands; 4 Functional Genomics Section, Center for Neurogenomics and Cognitive Research (CNCR), VU University, Amsterdam, The Netherlands; National Institute of Child Health and Human Development/National Institutes of Health, United States of America

## Abstract

**Background:**

The variance explained by genetic variants as identified in (genome-wide) genetic association studies is typically small compared to family-based heritability estimates. Explanations of this ‘missing heritability’ have been mainly genetic, such as genetic heterogeneity and complex (epi-)genetic mechanisms.

**Methodology:**

We used comprehensive simulation studies to show that three phenotypic measurement issues also provide viable explanations of the missing heritability: phenotypic complexity, measurement bias, and phenotypic resolution. We identify the circumstances in which the use of phenotypic sum-scores and the presence of measurement bias lower the power to detect genetic variants. In addition, we show how the differential resolution of psychometric instruments (i.e., whether the instrument includes items that resolve individual differences in the normal range or in the clinical range of a phenotype) affects the power to detect genetic variants.

**Conclusion:**

We conclude that careful phenotypic data modelling can improve the genetic signal, and thus the statistical power to identify genetic variants by 20–99%.

## Introduction

The aim of genome-wide association studies (GWAS) is to find genetic variants that are associated with variation in a phenotype of interest or with increased risk of disease. GWAS have successfully located genetic variants for medical and psychiatric disorders [1]–[7], but the variance explained collectively by these genetic variants is small compared to the heritability estimates obtained in family studies. For instance, the heritability (h^2^) of ADHD is estimated at ∼76% [8], yet the variants identified in GWAS explain only ∼1% of the variance [9].

This issue of ‘missing heritability’ [10] is viewed as a serious problem in GWAS. The majority of explanations and solutions put forward to date concern genetic issues, such as genetic coverage, penetrance, copy number variation, epistasis, gene-environment interaction, epigenetics, genetic heterogeneity, rare variants, limited genetic variation in the study sample, genotyping errors, incomplete LD between the marker SNPs and the causal variants, and parent-of-origin effects [10]–[17]. However, at least as important to the detection of genetic variants for complex traits is the way complex traits are measured, and the phenotypic information is modelled. Researchers are generally aware of the theoretical importance of unbiased, reliable and replicable measurement, but the issue of modelling of phenotypic information has not enjoyed much attention in GWAS. This neglect is unfortunate because, as we demonstrate here, measurement problems can diminish the association signal, and thus hamper the detection of genetic variants. Using simulation studies, we show that three phenotypic measurement issues - phenotypic complexity (*Study 1*), measurement bias (*Study 2*), and phenotypic resolution (*Study 3*) - provide additional viable explanations of the missing heritability.

Many psychological, psychiatric, and other (medical) traits cannot be observed directly, and are therefore measured using psychometric or diagnostic instruments. Such traits are denoted as *latent* variables [18] to emphasize that the trait itself is an unobservable attribute (e.g., ‘intelligence’, ‘depression’, ‘asthma’), which plays a causal role in shaping observable behaviour, such as scores on an IQ test, or the presence of depressive or asthma symptoms. In the studies presented below, we adhere to this standard latent trait perspective, as this is the prevailing view on phenotypes in behavioural genetics. We illustrate how advanced modelling of the phenotypic information can lead to identification of genetic variants that may otherwise go undetected. In the studies below, we used R [19] to simulate data, and used R or Mx [20] for data analysis. All simulations scripts are available online (Scripts S1).

## Materials and Methods

### Study 1: phenotypic complexity

Psychometric or diagnostic instruments are used to measure latent traits. While the aim of many GWAS is to detect genetic variants that cause individual differences in a given latent phenotype, actual GWAS analyses often rely on a sum-score operationalization. A sum-score is simply the sum of the responses to the test's items or symptoms. In the case of diagnostic instruments, the sum-score usually consists of the number of endorsed symptoms, and is often dichotomized to create an affection-status dichotomy, which serves to distinguish cases and controls. This dichotomized sum-score is used in GWAS to examine allele frequency differences between cases and controls.

**Figure 1:**
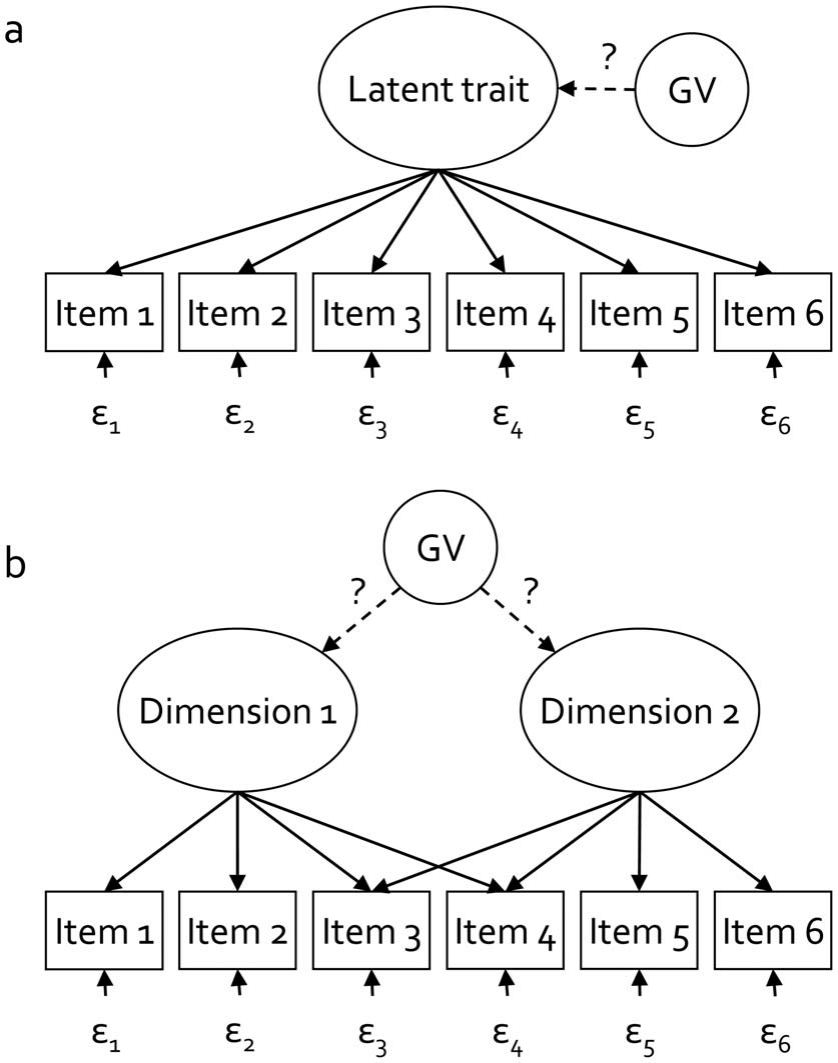
Uni- or multidimensionality in latent factor models. Figure 1a shows a graphical representation of a unidimensional factor model: one latent factor affecting the scores on 6 items. The effect of the genetic variant (GV) on the items scores is indirect, running via the latent trait. Often, however, scores on a test are not determined by one, but by multiple latent traits, or sub-dimensions of a latent trait, such as depicted in Figure 1b, where the scores on the first two items are determined by dimension 1, the scores on the last two items by dimension 2, and the scores on the middle items by both dimensions of the latent trait. Genetic association studies are complicated by this multidimensionality, because it is unknown beforehand whether genetic variants affects either or both dimensions.

Many latent traits of interest are essentially multidimensional (Figure 1), and instruments are designed to measure the distinct dimensions. For example, multidimensionality of cognitive ability is evident in the 14 subscales of the Wechsler Adult Intelligence Scale [21], which measure four correlated latent variables: Verbal Comprehension, Perceptual Organisation, Working Memory and Perceptual Speed. Twin and family studies have shown that this phenotypic multidimensionality is mirrored by genetic multidimensionality: genetic influences contribute to the phenotypic correlations between the dimensions, but dimension-specific genetic effects are also substantial [22]–[26], [ but also 27]. Similarly, the multiple dimensions describing ADHD-related childhood behavioural problems (e.g., hyperactivity, cognitive problems, attention problems, impulsivity, social problems) are all represented in instruments such as the *Child Behavior Check List* [28]. Again, these phenotypic dimensions are genetically correlated, but also show dimension-specific genetic effects [29]–[30].

Notwithstanding the complexity of traits, overall sum-scores, calculated across all subscales or dimensions, commonly feature as the dependent variable in GWAS. However, sum-scores are ‘sufficient statistics’, i.e., exhaustively summarizing all information available in the individual items or symptoms, only if the following three conditions hold [31]–[32]:

1. the test is unidimensional: only one latent trait underlies the scores on the set of items (or symptoms), and conditional on this latent trait, the items are statistically independent;
2. the expected values of the item responses have identical functional relations to the latent trait, operationalized as equal factor loadings in linear latent factor models for continuous items, or equal discrimination parameters in item-response theory models for dichotomous items;
3. in the linear latent factor model, the variance not explained by the latent trait (residual variance) is equal for all items.

If any these conditions are violated, sum-scores no longer optimally represent the latent trait, and the use of sum-scores may decrease the power to detect genetic variants for that trait, compared to more advanced phenotypic models, such as latent factor models. In family-based heritability studies, the unwarranted use of sum-score can result in the attenuation of phenotypic correlations between family members [33]–[34], but the effect of the use of sum-scores has not been studied in the context of GWAS. In *Study 1*, we investigated how the unwarranted use of sum-scores can affect the power to detect genetic variants in GWAS.

#### General settings Study 1

The following settings were used in all simulations in *Study 1*, unless stated otherwise. We assumed a measurement instrument including 6 standard normally distributed (∼N(0,1)) test items. These items were indicators of one or more latent factors. We simulated a biallelic genetic variant (A-a), with allele frequencies .5/.5, and coded the genotypes −1 (aa), 0 (Aa), and 1 (AA). The genetic variant explained 1% of the variance in one of the latent factors (note that this genetic variant is related to the test items but only via the latent factor). Conditional on this genetic variant, the factors were all standard normally distributed (∼N(0,1)). As the items were standardized, the residual variances of the items can be calculated as 1−λ_ij_^2^*(Ψ_j_+(β_j_^2^*.5)), where λ_ij_ is the factor loading of the i^th^ item on the j^th^ factor, Ψ_j_ is the variance of the j^th^ factor conditional on the genetic variant (1), β_j_ is the weight of the regression of the j^th^ latent factor on the genetic variant, and .5 is the variance of the genetic variant (given the present coding of the three genotypes and allele frequency of .5).

We simulated data for 1200 subjects using exact data simulation [35]. In each simulation study, we compared the power in two designs to detect the genetic variant. First, we added the scores on the items to form the sum-score, and then regressed the sum-score directly on the genetic variant (the ‘sum-score model’). Second, we modelled the data according to the *true* model, i.e., the model used to simulate the data, and regressed the latent factor on the genetic variant (the ‘true model’). To get an indication of the statistical power to detect the genetic variant, we fixed the regression coefficient to zero in both models, to obtain the increase in χ^2^ (i.e., the likelihood ratio test with 1 degree of freedom, df).

#### Violation unidimensionality

**Figure 2:**
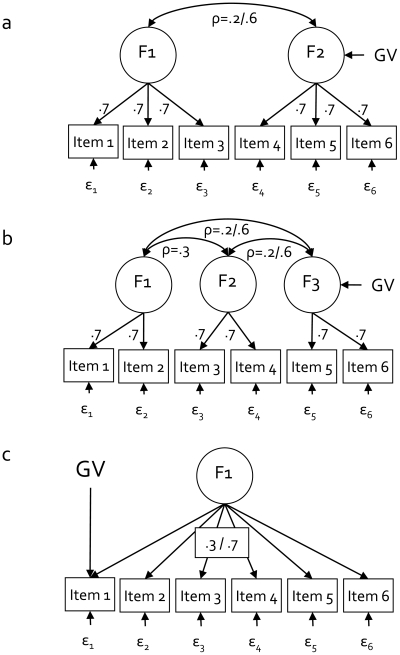
Factor models used for simulation in Studies 1 and 2. *Study 1:* Data were simulated according to a 2-dimensional (Figure 2a) or 3-dimensional (Figure 2b) latent factor model, with factorial correlations ρ ranging between .2 and .6, and factor loadings fixed to .7. *Study 2:* Data were simulated according to a 1-factor model (Figure 2c), with all items having either a weak or a strong relation to the latent factor (factor loadings of .3 or .7, respectively). The genetic variant (GV) affected the first item only.

To investigate the question of how violation of the unidimensionality condition affects the power to detect genetic effects in the sum-score model, we simulated data according to a two- and a three-factor model. In the two-factor model (Figure 2a), items 1 to 3 loaded on the first factor, and items 4 to 6 loaded on the second factor. The correlation between the two factors was .2 or .6, and the genetic variant affected the second latent factor only. In the three-factor model (Figure 2b), items 1 and 2 loaded on the first latent factor, two items 3 and 4 on the second factor, and items 5 and 6 on the third factor. The correlation between the first and second factor equalled .3, but the correlation between the third factor and the other two factors was .2 or .6, and the genetic variant affected the third latent factor only. In both models, all factor loadings equalled .7.

#### Violation equal factor loadings

To find out how the violation of equal factor loadings affects the power to detect genetic effects in the sum-score model, we simulated data according to a unidimensional measurement model, comprising 6 or 12 items. In simulation 1, half of the factor loadings equalled .3 (unreliable items), and half to .9 (reliable items). In simulation 2, ^1^/_3_ of the factor loadings equalled .5, ^1^/_3_ equalled .7, and ^1^/_3_ equalled .9. So as not to violate the condition of equal residual variances, we set all residual variances to .6, irrespective of the factor loadings.

#### Violation equal residual variance

To investigate how the violation of equal residual variances affects the power to detect genetic effects in the sum-score model, we simulated data according to a unidimensional factor model, with 6 or 12 items. Factor loadings of all items equalled .6. In simulation 1, half of the residual variances equalled .64, and the other half 1.64 (1 SD higher). In simulation 2, half of the residual variances equalled .64, and half 2.64 (2 SD higher).

### Study 2: Measurement bias

In comparing groups with respect to the latent trait, one needs to establish that the test used to measure the trait is not biased, i.e., that the instrument is ‘measurement invariant’ (MI) with respect to group [36]–[37]. MI implies that the test measures the same latent trait in the different groups or samples. For example, imagine a test measuring psychometric IQ. Subjects who have the same latent intelligence should have equal probability of answering the items on this test correctly. If the test is not MI with respect to, say, sex, men and women with the very same latent intelligence have systematically different probabilities of answering one or more items on that test correctly. For instance, items requiring mechanical knowledge may reflect sex differences in interest and experience rather than sex differences in intelligence. As a consequence, the sex differences in observed test scores can not be taken as indicative of sex differences in latent intelligence, and such bias-related variation in observed test scores may suppress variation due to genetic variants.

In the linear factor model, MI holds if the following four conditions are satisfied. First, the factor structure is the same in all samples (the configuration of factor loadings is identical: ‘configural invariance’). Second, the factor loadings are equal over samples (‘metric invariance’). Third, the mean differences between samples on the level of the observed test items are fully attributable to mean differences between the samples at the level of the latent trait(s) (‘strong factorial invariance’). In combination with equal factor loading, this condition is satisfied if the intercepts in the regression of the observed item responses on the latent trait(s) are equal over samples. Fourth, the residual item variances (not explained by the latent trait(s)) are equal across samples (homogeneity of the residual variances, ‘strict factorial invariance’). Although heterogeneous residual variances do not invalidate the interpretation of observed mean differences in terms of latent trait mean differences, such heterogeneity may decrease the power to detect the effects of genetic variants in the combined sample.

Implicitly, the phenotypic measures used in GWAS are assumed to be MI across different samples (e.g., men-women, cases-controls, samples from different countries), but MI is rarely actually tested. Consequences of violations of MI have been studied in family-based heritability research [34], [38], but not in GWAS. Yet, MI is potentially important in GWAS, because information of samples is compared (case-control design) or combined (analysis of pooled raw datasets, i.e., mega-analysis).

Violations of MI with respect to the genetic variant itself are also possible. In GWAS, researchers assume that the genetic variant explains variance in the latent trait, and that the effect of the variant on individual items or symptoms is mediated by the latent trait (Figure 1a). It is however conceivable [39]–[40] that a genetic variant affects items or symptoms directly (Figure 2c). For instance, a genetic variant could relate to the ADHD symptom ‘fidgety’ but not to ADHD symptoms ‘temper outbursts’, ‘forgetful’, and ‘has lots of fears’. Similarly, variants could relate to visuo-spatial performance, but not to other cognitive abilities represented in intelligence tests, like memory and vocabulary. If genotype groups do not differ with respect to the latent trait (i.e., the genetic variant is not associated with ADHD or intelligence), but they do differ with respect to a specific symptom or ability, then this is a violation of MI with respect to the genetic variant. In *Study 2*, we investigated how the power to detect genetic variants is affected by all five violations of MI.

#### General settings Study 2

The following settings were used in all simulations presented in *Study 2*, unless stated otherwise. We assumed a measurement instrument including 6 standard normally distributed (∼N(0,1)) test items, influenced by one or more latent factors. We simulated a biallelic genetic variant, with allele frequencies .5/.5, and coded the genotypes −1, 0, and 1. The genetic variant explained 1% of the variance in one of the latent factors. Note that the genetic variant is related to the test items via the latent factor. Conditional on this genetic variant, the factors were all standard normally distributed (∼N(0,1)) (again, as all items were standardized, the residual variances of the items can be calculated as 1−λ_ij_^2^*(Ψ_j_+(β_j_^2^*.5)), where λ_ij_ is the factor loading of the i^th^ item of the j^th^ factor, Ψ_j_ is the variance of thej^th^ factor conditional on the genetic variant (1), β_j_ is the weight of the regression of the j^th^ latent factor on the genetic variant, and .5 is the variance of a genetic variant with allele frequencies .5/.5. We simulated data for two samples of N = 600 each using exact data simulation^34^. In each simulation study, we compared the power to detect the genetic variant between two designs: the ‘sum-score model’ (the sum-score calculated across all items is regressed on the genetic variant), and the ‘true model’ (the items are subjected to the model that was used to simulate the data, and the latent factor is regressed on the genetic variant). To get an indication of the statistical power to detect the genetic variant in the two designs, we studied the deterioration of the model fit, expressed as increase in χ^2^, when the association between the genetic variant and the operationalisation of the trait (sum-score or latent factor) was fixed to 0, i.e., a test with 1 degree of freedom (df).

#### Measurement invariance with respect to sample: configural invariance

A violation of configural invariance implies that the factor structure (i.e., the configuration of factor loadings) is not identical across samples. We simulated data for the first sample according to a 2-factor model, with items 1 and 2 loading on Factor 1, with factor loadings of .4, and .5, respectively, and items 3 to 6 loading on Factor 2, with loadings of .7,.6, .5, and .4, respectively. For the second sample, items 3 and 4 also loaded on the first factor, with loadings of .3 or .6, respectively. In both samples, Factors 1 and 2 correlated .3, and the genetic variant affected only the second factor.

#### Measurement invariance with respect to sample: metric invariance

A violation of metric invariance implies that the factor loadings are not equal over samples. In practice, such a violation may concern only a few of the factor loadings. We simulated data in two samples according to a 1-factor model. In the first sample, all loadings equalled .5. In the second sample, the loadings of items 1 and 2 were either.3 or .9. Irrespective of the factor loadings, the residual variances of all items in both samples equalled .747 (i.e., given a factor loading of .5 and the GV, the variance of the indicator was 1).

#### Measurement invariance with respect to sample: strong factorial invariance

Strong factorial invariance implies that differences between samples in expected values of observed scores are not solely indicative of differences between samples in latent factor scores. If for some items, the expected observed item score differences can not be explained by differences on a latent level (because the observed differences are too small, or too large, given the difference in latent factor means between the samples), then these items are considered to be biased. In both samples, we simulated data according to a 1-factor model with factor loadings equal to .5. In the first sample, the means of all items and the latent factor were fixed to 0. In the second sample, all means were fixed to 0, except the means of the first two items, which varied from .1, to .5, to 1, i.e., the second sample scored .1, .5 or 1 SD higher on these items than the first sample, even though both samples had equal latent factor means. In terms of the factor model, this setup implies that the intercept of items 1 and 2 differ across the samples.

#### Measurement invariance with respect to sample: strict factorial invariance

Strict factorial invariance implies that the factor structure, factor loadings, item intercepts and residual item variances are equal across samples. If the factor loadings and factorial variances are equal across samples, but the residual variances are not, then this implies that the percentage of variance explained by the factor in the items is not equal across samples, and thus that the reliability of the items is not the same (in the context of the factor model, the reliability of an item is defined as the ratio of the variance explained by the factor and the total variance of the item). In the factor model, the item variance is decomposed in to a part due to (explained by) the common factor(s) and a residual part. Because the residual variances are separated from the latent factor, differences between samples in residual variances (i.e., violations of strict factorial invariance) are not expected to greatly affect the power to detect a genetic variant if the genetic effect is directly on the latent factor. To investigate this we simulated in two samples data we simulated data according to a 1-factor model, with factor loadings for all items fixed to .5, and all means fixed to 0. In the first sample, residual variances equalled .747, while in the second sample the residual variances of the first two items equalled this value plus .5, 1, or 2, i.e., these residual variances were .5, 1 or 2 SD larger.

#### Measurement invariance with respect to the genetic variant

A direct relation between a genetic variant and an item (or symptom, or subtest; Figure 2c), rather than via the latent factor, can be viewed as a violation of MI. MI with respect to the genetic variant implies that observed differences between the genotype groups are interpretable in terms of differences in the latent trait. If the three genotype groups (i.e., aa, Aa, and AA) do not differ with respect to the latent trait (i.e., the variant is not associated with the latent trait), but they do differ with respect to any item (i.e., direct relation between the variant and the item), then this item is considered biased with respect to the genetic variant.

To find out how violations of MI with respect to the genetic variant itself affect the power to detect that variant, we again assumed a measurement instrument including 6 items, and simulated data according to a 1-factor model for N = 1200 subjects. We now introduced the genetic effect directly on only the first item, not on the factor (Figure 2c). The genetic variance explained 1% of the variance in the first item. Allele frequencies were set to .5/.5. The factor loadings of all items equalled either .3 or .7, such that the sum-score could serve as a sufficient statistic (with respect to the phenotypic part of the data). We studied the power to detect the genetic variant in 4 designs: 1) the sum-score model, 2), the 1-factor model with the genetic effect modeled on the latent factor, 3) the item model, in which only the first item is regressed on the genetic variant (i.e., information from the other 5 items is discarded), and 4) the true model, i.e., a 1-factor model with the genetic effect directly on the first item only (Figure 2c).

### Study 3: Phenotypic resolution

The statistical power to detect a genetic variant depends on the reliability of the phenotypic instrument. Test-reliability is often expressed as some approximation of the ratio of the variance attributable to the latent trait of interest (systematic variance) to the total variance of the measure (including unsystematic and error variance). For example, if a sample size of N≈780 is required for a power of 80% to detect a genetic variant that explains 1% of the variance in the error-free latent trait, then N≈1300 is required to achieve the same power if the psychometric instrument has a reliability of .7. In this conceptualisation, the reliability of a test is stable across the entire phenotypic range of a certain population. However, the issue of reliability, however, can be conceptualized as one of ‘resolution’ [41], and the resolution of a test is not usually stable across the entire phenotypic continuum.

**Figure 3:**
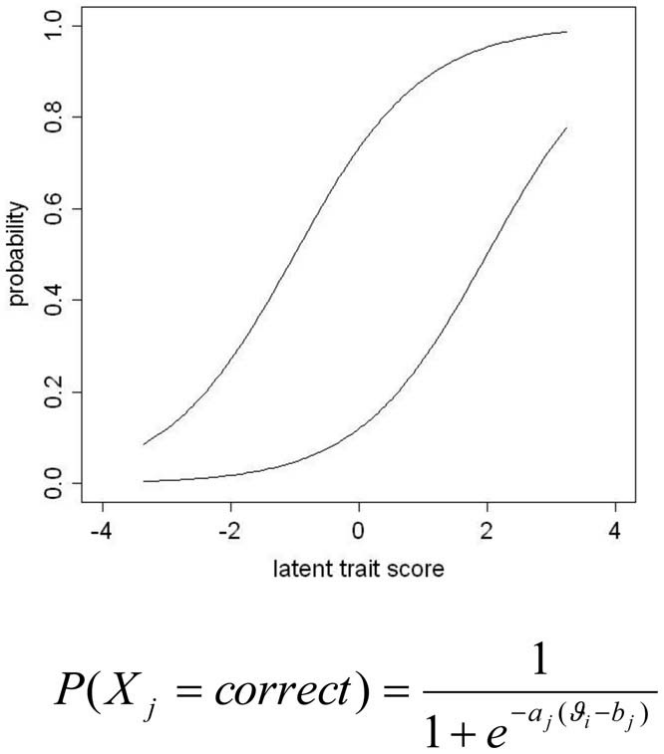
Item characteristic curves in a 2-parameter Item Response Theory (IRT) model. Figure 3 shows the item characteristic curves of two items describing the probability of answering the items correctly (affirmatively) given one's latent trait score *θ*. The first item (left) has difficulty parameter *b* = −1, i.e., subjects with (standardized) latent trait score equal to *ϑ* = −1 have 50% probability to endorse this item, while subjects with latent trait score *ϑ* = 2 endorse this item with 95% probability. The second item (right) has difficulty parameter b = 2, i.e., subjects with latent trait score *ϑ* = 2 have 50% probability to endorse this item, while subjects with latent trait score *ϑ* = −1 only have 5% chance. Both items have discrimination parameter *a* = 1 (i.e., equal slopes), determining the degree to which a given item discriminates between subjects with different latent trait scores. In contrast to items with low discrimination parameters (flat slopes), items with high discrimination parameters (steep slopes) discriminate well between subjects whose latent trait scores lie closely together within a narrow range. The 2-parameter logistic model [44], [45] can be used to calculate for every subject *i* the probability of endorsing an item X*_j_* given this item's discrimination parameter *a_j_* and difficulty parameter *b_j_*.

The resolution of a test is defined as its ability to resolve phenotypic differences between individuals. Ideally, a test should contain items with difficulty parameters well distributed across the full range of the latent phenotype and with good resolution (Figure 3).

In practice, however, tests are usually tailored to a certain target population. For instance, most cognitive tests are designed to resolve individual differences in the middle or ‘normal range’, and therefore include items with intermediate difficulty. In contrast, measures of psychopathology, such as depression, aim to differentiate between subjects who do, and who do not, qualify for clinical diagnosis, and therefore comprise relatively extreme items. Since items like “I think of suicide everyday” will not be endorsed by many people from the general population, this item's ability so resolve individual differences in depression-related behaviour in the general population is limited. In a clinical subsample, however, this same item may be very informative as it distinguishes individuals suffering from mild or severe depression.

Ideally, a test should have high resolution throughout the expected phenotypic range that characterizes the population of interest. Because the range of interest in GWAS often spans the normal/unaffected as well as the affected, and thus is necessarily wide, there is no guarantee that the resolution of the psychometric instrument is sufficient throughout the entire range of interest. In family-based heritability studies, insufficient resolution can result in underestimation of h^2^ [e.g., 42], and spurious gene-environment interaction [43]. In *Study 3*, we investigated how resolution affects the power to detect genetic variants in GWAS, and where on the latent phenotype continuum the test should have good resolution to maximize the probability to detect genetic variants.

#### General settings Study 3

In Item Response Theory (IRT), discrete test items are characterized by 2 parameters: a difficulty parameter and a discrimination parameter (Figure 3) [44]–[45]. An item's discrimination parameter, corresponding to the slope of the item characteristic curve, is informative concerning the item's ability to resolve individual differences (i.e., discriminate between subjects with different latent trait scores), with high parameters indicating that the item discriminates well between subjects, whose latent phenotype scores lie closely together. The difficulty parameter of an item corresponds to the position on the latent phenotype continuum where the resolution of the item is maximal. If an item has low (high) difficulty, then the item resolves individual differences in the lower (higher) range of the latent phenotype continuum.

Using IRT as theoretical basis of our simulations, we simulated 27 items across the entire phenotypic continuum, with difficulty parameters ranging from −4 to 3.8, with steps of .3 (assuming a standard normal latent trait), and fixed discrimination parameters of 1 (i.e., Rasch model). Specifically, the difficulty parameter of the first item equalled −4, so that subjects with a latent trait score of −4 have 50% chance to answer this item correctly. The difficulty parameter of the 15^th^ item equaled .02, so that subjects with a latent trait score of .02 have 50% chance to answer this item correctly, etcetera. As all items had equal discrimination parameters, a sum-score would be a sufficient statistic for this test.

We used the 27 items to compose 5 separate test instruments: 1) a comprehensive instrument including all 27 items, 2) an instrument including only the 9 middle items (difficulty parameters −1.3, −1.0, −0.7, −0.4, −0.1, 0.2, 0.5, 0.8, and 1.1), corresponding to a test constructed to measure behavior within the normal range, 3) an instrument including 9 high extreme items (difficulty parameters 1.4, 1.7, 2.0, 2.3, 2.6, 2.9, 3.2, 3.5, and 3.8), corresponding to a diagnostic test constructed to measure extreme behavior, 4) an instrument including 9 items covering the entire continuum (difficulty parameters −4.0, −3.1, −2.2, −1.3, −0.4, 0.5, 1.4, 2.3, and 3.2), and 5) an instrument including 5 low-extreme items, and 4 high-extreme items (difficulty parameters −4.0, −3.7, −3.4, −3.1, −2.8, and 2.9, 3.2, 3.5, 3.8).

We simulated 71 genetic variants for N = 2500 subjects: 50 with small effect (genotypic value = .01), 20 with a larger effect (genotypic value = .05), and 1 with a still larger effect (genotypic value = .1). Frequencies of alleles A and a were both .5 for all 71 variants. We then created individual subject's latent phenotype scores by summing the genotypic values associated with the individual's genotypes on all 71 variants. Variation in the latent trait scores was thus solely due to the effects of the 71 genetic variants. We then standardized these latent trait scores to z-scores. The genetic variants with small, medium and large effect explained ∼.05%, ∼2.5% and ∼11% of the variance in the standardized latent trait score, respectively. We used the standardized latent trait scores to calculate, for every person, the probability of answering each of the 27 items correctly, using the formula for the 2-parameter IRT model [44], [45]

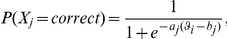
where X_j_ is the score on item *j*, *a*_j_ and *b*_j_ are the discrimination parameter and the difficulty parameter for the *j* ^th^ item, respectively, and θ_i_ is the standardized latent trait score of the *i* ^th^ person (note that this formula reduces to the Rasch model as all *a*_i_'s are fixed to 1 in our simulation).

Based on these probabilities, we created item scores coded 0 (incorrect) or 1 (correct) for every subject, and calculated the 5 sum-scores for each of the 5 instruments (e.g., a sum-score based on all 27 items, a sum-score based on the 9 middle items, etc). We related these 5 sum-scores to the first genetic variant with small effect (genotypic value of .01, explaining about .05% of the variance in the trait under study) in two designs: 1) a population-based sample design, with 2500 subjects randomly selected from across the entire trait continuum, and 2) a selected-samples design, with 1250 subjects with phenotype scores in the top 5% range (‘cases’) and 1250 subjects with phenotype scores in the 0–95% range (random selection; ‘controls’).

In each design, we related the 5 different sum-scores to the genetic variants using a one-way ANOVA with three groups (i.e., the genotype groups aa, Aa, and AA), yielding 5 different p-values. As the creation of the test scores was based on a stochastic process, this entire simulation was repeated 10.000 times.

## Results

### Study 1: phenotypic complexity

**Figure 4:**
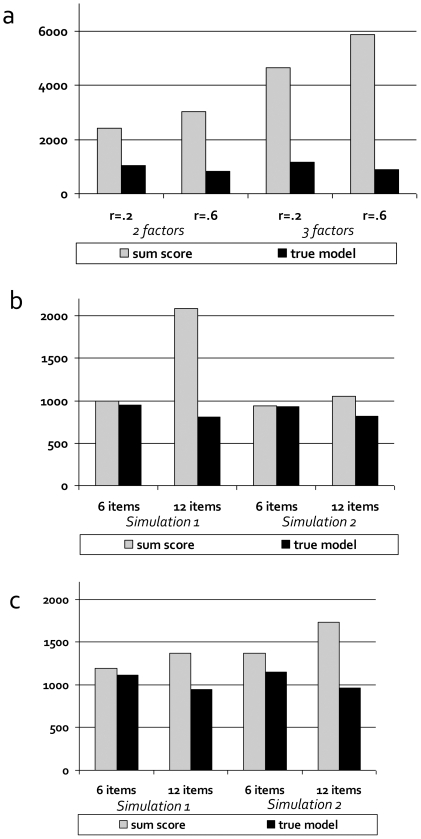
The power to detect genetic variants is lower if sum-scores are not sufficient statistics (results Study 1). Figures 4a–c show the sample size required for a power of 80% to detect a genetic variant (GV) that explains 1% of the variance on the latent level, using either the sum-score model or the true latent factor model. Figures show the effects of violation of unidimensionality (Figure 4a), violations of equal factor loadings (Figure 4b), and of violations of equal residual variances (Figure 4c).

Figure 4 summarize the effects of the violations of the three conditions required for sum-scores to be sufficient statistics (unidimensionality, equal factor loadings, and equal residual variances) on the power to detect genetic effects (see also Tables S1, S2, S3, S4, S5, S6, S7).

Note first that if the three conditions are satisfied, the power of the sum-score model is identical to the power of the true factor model (not shown). Second, we found that, when genetic association analysis is conducted on sum-scores, while the unidimensionality condition is violated, the power to detect genetic variants that are specific to one dimension is substantially decreased, especially when the number of dimensions increases, and the correlations between dimensions increase (Figure 4a). This is because a sum-score mainly summarizes the variance *shared* by the factors (i.e., shared by the underlying items). Genetic effects that are specific to one of the factors, i.e., are related to the variance that is not shared between the factors, will be harder to detect when the variance shared between the factors is large and dominates the sum-score. Specifically, in our simulation, the power of the sum-score models was only 33–43%, and 19–27% of the power of the true latent factor model for the case of 2 and 3 latent dimensions, respectively. Given 6 items, N = 1200, and 10 genetic variants explaining 1% of the variance each in one dimension of a two-dimensional trait, the probability to detect 6 or more of these variants would be >.95 under the true latent factor model, and <.20 when using the sum-score model (with the exact probability depending on the correlation between the factors). For a three-dimensional trait, these probabilities are >.90 and <.01, respectively. Only in the specific case that the genetic variant affects all latent dimensions to exactly the same extent, is the power to detect the genetic variant approximately equal for the sum-score model and the factor model (see Tables S3, S4). Third, we found that when the condition of equal factor loadings is violated (Figure 4b), the power to detect genetic effects on sum-scores is decreased compared to the true latent factor model, which accommodates unequal factor loadings. The difference in power is larger when the factor loadings are more variable, and the number of items increases. Specifically, for 6 items, the power of the sum-score model was 86–93% of the power of the true latent factor model, depending on the differences between the factor loadings. For 12 items, the effect of the violation of equal factor loadings was more pronounced, with the power of the sum-score model being 39–78% of the power of the true latent factor model. Given 6 items, N = 1200, and 10 genetic variants explaining 1% of the variance each on the level of the latent trait, the probability to detect 6 or more of these variants would be high for both the sum-score model and the factor model (.97 and .98, respectively) even if the factor loadings show considerable differences (Simulation 1). Yet for 12 items, the probabilities would be .29 and .99 for the sum-score model and the factor model, respectively, suggesting increasing misfit and increasing loss of power with increasing number of items. Fourth, we found that if the condition of equal residual variances is violated, the power to detect genetic effects on sum-scores is decreased compared to the true latent factor model (Figure 4c). The difference in power becomes larger when the residual variances are more variable, and the number of items increases. Specifically, the power of the sum-score model was 78–98% of the power of the true latent factor model when the residual variances differed about 1 SD, and became 56–82% when the residual variances differed 2 SD. Given 6 items, N = 1200, and 10 genetic variants explaining 1% of the variance each on the level of the latent trait, the probability to detect 6 or more of these variants is quite comparable for the sum-score model and the true latent factor model (.88 and .93, respectively), if the difference in residual variance is 1 SD, but less so if the difference is 2 SD (.78 versus .91, respectively). For 12 items, the probabilities are .78 and .98 for 1SD, and .51 and .98 for 2 SD, for the sum-score model and the factor model, respectively. This again suggests increasing misfit and increasing loss of power with increasing number of items.

In sum, the simulations of Study 1 show that when conditions for calculating sum-scores are violated, proper phenotypic modelling, instead of the use of simple sum-scores, will generally confer appreciable increases in the power detect genetic variants.

### Study 2: Measurement bias

**Figure 5:**
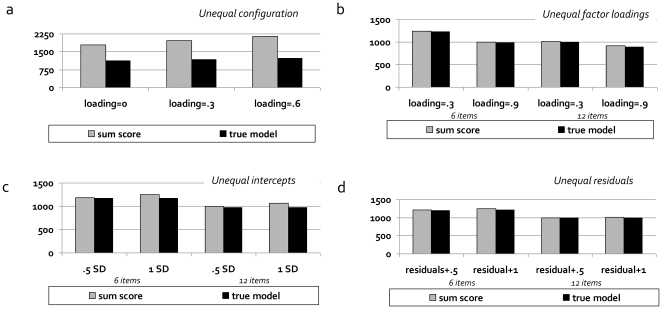
The power to detect genetic variants is slightly affected by violations of measurement invariance with respect to sample (results Study 2). In the case of continuous items, measurement invariance (MI) with respect to sample holds if 1) the factor structure is identical across samples (‘configural invariance’; Figure 5a), 2) the factor loadings relating the observed items to the latent trait(s) are identical across samples (‘metric invariance’; Figure 5b), 3) mean differences between samples on the individual items are attributable to mean differences at the latent level (‘strong factorial invariance’; Figure 5c), and 4) the variance in item scores not explained by the latent trait(s) is equal across samples (‘strict factorial invariance’; Figure 5d). We simulated these four types of violations of MI, and analyzed the data using either the sum-score model or the true latent factor model. Figures 5a–d show the sample size required for a power of 80% to detect a genetic variant that explains 1% of the variance on the latent level under these four different kinds of violations of MI.

Figure 5 shows the results of four types of violations of MI with respect to sample. In each simulation we compared the power to detect the genetic variant in the sum-score model to the power in the true latent factor model, in which we accommodated the violations. To test the effect of these violations in its purest form, we chose all simulation settings such that a sum-score could in principle serve as a sufficient statistic, except for the violation of interest. In this ideal situation, cross-sample violations of equality of factor loadings, equality of residual variances, and equality of observed item means hardly affected the power to detect genetic variants (Figure 5b–d). Configural invariance, however, necessarily implies a multi-dimensional model, so sum-scores are never sufficient statistics (see *Study 1*), and the power to detect genetic variants under the sum-score model is always lower than the power under the true latent factor model. However, comparisons within models (loading = 0 versus loading = .3 or .6) show that violations of configural invariance affect the power in both models, but more so in the sum-score model (Figure 4a). Actually, whether the power to detect the genetic variant reduces or indeed increases as a result of violations of configural invariance, depends on whether the cross-loadings concern the latent factor that is associated with the variant (increase in power) or the latent factor that is not associated with the variant (decrease in power, see Figures S1, S2 and Tables S8, S9, S10, S11, S12, S13, S14, S15).

**Figure 6:**
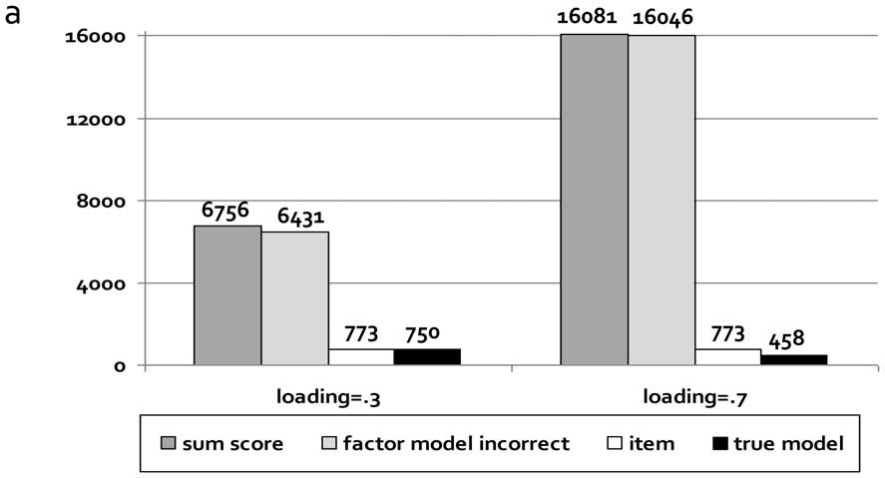
The power to detect item-specific genetic variants greatly depends on the fitted phenotypic model (results Study 2). When the effect of a genetic variants does not run via the latent factor but is directly on, and specific to, one of the items (as illustrated in Figure 2c), we speak of violations of measurement invariance with respect to the genetic variant itself. Figure 6 shows the sample size required for a power of 80% to detect such an item-specific genetic variant that explains 1% of the variance in the first item only.

If the effect of a genetic variant is specific to a certain item or symptom, rather than affecting all items via the common factor (i.e., violation of MI with respect to the genetic variant, Figure 2c), then the likelihood to detect that variant is greatly diminished if its effect is modelled on the sum-score or directly on the latent factor (‘incorrect latent factor model’), compared to the correctly specified latent factor model and a model in which that specific item/symptom is directly regressed on the genetic variant (Figure 6, Table S16). Sample sizes required for a power of 80% increase from ∼800 subjects in the correctly specified models to over 6,000 or even 16,000 subjects in incorrectly specified models. Practically, given 6 items, N = 1200, and 10 genetic variants explaining 1% of the variance each in the first item only, the probability to detect 6 or more of these genes would be >.99 for the correctly specified latent factor model and the model in which the specific item is directly regressed on the genetic variant. The chance to detect 6 or more of these genes is dramatically decreased to <.01, if the sum-score model or the incorrectly specified latent factor model is used.

In sum, the simulations of Study 2 show that the presence of measurement bias constitutes a threat to the success of GWAS, but primarily when the bias concerns the genetic variant itself: in that case, the power of misspecified models is considerably lower.

### Study 3: Phenotypic resolution

The results of the 10.000 simulations are summarized in Table 1 (see Figures S3, S4 for the distributions of the p-values, and Figures S5, S6 for the Test Information Curves of both designs, as well as Table S17 for the results of similar simulations with a genetic variant explaining .6% of the variance, which showed a very similar pattern of results). The results in Table 1 show that in both the selected-samples design and the population-based design, the genetic variant is detected most often, when the test including all 27 items is used. Of the subscales including only 9 of 27 items, the scale including 9 middle items conferred the greatest power to detect the genetic effect, irrespective of the study design. Given α = .05, the power of this subscale is 77 to 90% of the power of the full scale for the population-based design and the selected-samples design, respectively. The power of the subscale including 9 high-extreme items is only 52 to 81%. Note also that the scale including 9 items across the entire scale is usually more powerful than the scale including 9 extreme items only. The scale including 5 low and 4 high extreme items is the least powerful in both designs. Practically, this means that in a population-based design, N = 2500, α = .05, and 10 genetic variants explaining only .1% of the variance each, the probability to detect 5 or more of these variants would be .13, .07,.01, .02 and .0004 for the test including 27 items, 9 middle items, 9 extreme items, 9 items across the scale, and the scale including 5 low and 4 high extreme items, respectively. For the selected-samples design, the probabilities are considerably higher: .55, .41, .29, .27, and .02, respectively.

**Table 1:** Results simulation study (Nsim = 10.000) into the power to detect a genetic variant explaining .1% of the variance with 5 differently constructed phenotypic instruments (an complete scale with 27 items, a subtest with the 9 middle items, a subtest with 9 items selected to cover the entire continuum, a subtest with 5 low-extreme and 4 high-extreme items, and a subtest with 9 high-extreme items) in two designs: a population design (N = 2500) and a selected-samples design (1250 extreme subjects and 1250 subjects from the normal range).

	α = .05				α = .01				α = .001			
	Population		Selected samples		population		Selected samples		population		Selected samples	
	#hits	Ratio	#hits	Ratio	#hits	ratio	#hits	Ratio	#hits	ratio	#hits	ratio
All 27 items	3763		5692		1741		3324		524		1276	
9 middle items	3289	.87	5119	.90	1477	.85	2780	.84	406	.77	985	.77
9 high extreme	1967	.52	4629	.81	706	.41	2340	.70	143	.27	768	.60
9 items across the scale	2606	.69	4546	.80	1009	.60	2358	.71	246	.47	780	.61
5 low-extreme+4 high-extreme	1171	.31	2589	.45	362	.21	1030	.31	56	.11	224	.18

Note. #hits denotes the number of p-values<α = .05, α = .01, or α = .001, respectively. Ratio denotes the % of hits that the 4 subscales pick up, compared to the full instrument including all 27 items.

Of the subscales, the scale including 9 middle items always performs best in the context of GWAS because the variation in test scores on this scale ‘matches’ the expected genetic variation. Specifically, cases (i.e., individuals with high latent trait scores), will endorse (almost) all middle items, such that the variation in their test scores is low. In contrast, the scores of ‘controls’ (i.e., individuals with latent trait scores representative of the ‘normal population’) are more variably on this scale, as they sometimes endorse items, but sometimes do not. In addition, given the common-trait-common-variant model, cases more often carry one or two (but not 0) copies of the detrimental allele, so variability in genotypes is lower in this group, compared to the genetic variability in subjects representing the normal population. For the test including 9 middle items, the variation in test scores (high in controls, low in cases) thus matches the genetic variation (high in controls, low in cases). In contrast, on the test including 9 extreme items, cases will show variability in test scores, while controls will hardly ever endorse these extreme items. Consequently, the variation in phenotypic scores on this subscale (low in controls, high in cases) does not match the genetic variation (high in controls, low in cases).

In sum, the results of Study 3 underline the importance of choosing phenotypic measurement instruments that resolve individual differences specifically in the part of the study population, where the genotypic variance is expected to be largest.

## Discussion

Our three simulation studies suggest that at least part of the missing heritability problem of complex phenotypes may originate in misspecification of the phenotypic model. The three phenotypic measurement issues that we consider can all strongly influence the genetic signal, and thus the power to detect genetic variants, and the appraisal of the associated effect sizes. Simulation results presented in Studies 1 and 2 suggest that re-analysis of available genotype-phenotype data is likely to identify additional genetic variants when the multi-dimensionality of the phenotype, and the possibility of genetic effects being specific to certain phenotypic dimensions or items, are taken into account. These re-analyses require the availability of phenotypic information on the level of individual items or questions. Such detailed information, at present only scarcely available, should be made accessible in public genotype-phenotype databases. Relevant for future research is our finding in Study 3 that the power to detect genetic variants improves if the trait of interest is measured using phenotypic instruments that resolve individual differences in those subpopulations, where the genotypic variance is assumed to be largest.

More sophisticated modelling of phenotypic information in the context of genetic association studies may greatly enhance the power to detect genetic variants, but creates its own demands. First, running genome-wide analyses on full factor models rather than on sum-scores is computationally more demanding. The use of cluster computers (and parallel software), which allow the parallel processing of information on multiple nodes at the same time, will overcome this disadvantage. Second, while establishing the link between a phenotypic sum-score and a genetic variant is straightforward, finding the ‘location’ of the genetic effect within a more complicated factor model (e.g., specific to one of the latent factors, or to one of the items) is potentially more complicated. So-called modification indices, used in factor analytic approaches to identify local misfit in larger models [46], may prove useful in guiding researchers towards the exact location. Finally, while determining the association with a sum-score or affection-status dichotomy requires one statistical test per genetic variant, establishing the association of a genetic variant in the context of a complex factor model may require multiple statistical tests per variant. The foreseen expansion of the multiple testing problem merits appropriate attention.

The heritabilities of potentially suboptimal phenotypic operationalizations (e.g., sum-scores) are often found to be considerable in family-based studies. How can this be reconciled with our finding that the use of these same sum-scores can seriously affect the power to detect the genetic variants underlying the high heritability estimates? The considerable heritability estimates observed for sum-scores reflect the concerted effect of all genetic effects on all individual items that are summed: not only the additive effects that are shared by all items, but also genetic effects that are specific to only one or a few of these items, dominance effects, epigenetic effects, epistatis, and effects of complete genetic pathways. Although heritability estimates based on sum-score operationalizations are expected to often be underestimated as well (see for example van den Berg and colleagues [47], who demonstrated that the sum-score of 7 attention problem items showed a heritability of 40%, while the heritability increased to 73% when it was estimated in the context of an item response theory model), they can still be considerable. A high heritability of the sum-score does, however, not guarantee that this operationalization is also useful for the detection of variant-specific effects, which are likely to be very small to begin with. The detection of variant-specific effects will suffer greatly when suboptimal operationalizations are used as the expected weak statistical association will be even weaker in the context of a poor and noisy operationalization.

We analyzed the impact of phenotypic measurement issues on gene finding from the standard continuous latent trait perspective. In this perspective, we accord the latent traits a causal status: one's position on the latent trait determines the probability of endorsing a given item or psychiatric symptom [18], [48]. This causal view of latent traits is consistent with the aim of GWAS to detect genetic variants that cause individual differences in operationalizations such as sum-scores. Of course, this causal interpretation of latent variables is based on a theoretical position, which itself is open to investigation. Recently, researchers in the field of psychology have challenged the existence of latent traits, specifically in the context of intelligence research [49]–[51] and psychiatric comorbidity research [40]. The proposed alternative phenotypic models, such as the network model, the mutualism model, and the index variable view, do not necessarily appeal to causal latent traits. Consequently, sum-score operationalizations, which in principle make sense under the latent trait model, do not do so under these alternative models. Deceptively, sum-scores can show considerable heritability under all these alternative phenotypic models [e.g. 49], even if the operationalization is not sensible from a phenotypic or genetic point of view. This implies that high family-based heritability is no guarantee that a sum-score is a reasonable proxy of a causal latent trait, or that a causal latent trait even exists.

In this paper, we focussed on phenotypic measurement issues that can be encountered in the gauging, operationalization, and quantification of complex phenotypes like psychological, psychiatric, and other (e.g., medical) traits. In this context of phenotypes whose operationalization and measurement poses a challenge, we showed that suboptimal operationalization and misspecifications of the phenotypic model can greatly dilute the genetic signal. The three measurement issues that we discussed do, however, not apply to phenotypes like height and weight (body mass index), whose actual measurement is simple, but whose considerable heritability also hitherto remains largely “missing” [but see 17]. We note that the genetic explanations of the missing heritability, which may apply to these simple-to-measure phenotypes (e.g., incomplete LD between markers and causal variants [17]), may apply equally to the psychometrically complex phenotypes. Clearly, with respect to their effect on power to detect genetic association with a complex phenotype, genetic issues discussed elsewhere [10]–[17] and the measurement issues discussed here are by no means mutually exclusive.

Irrespective of the phenotypic model of choice, optimized modelling of the phenotypic part of the genotype-phenotype data improves the power to detect genetic variants. Modern psychometrics [44]–[45] offers statistically and theoretically well developed methods, such as (genetically informed) latent factor models and Item Response Theory, for addressing the phenotypic measurement issues discussed here, and as such has the potential to contribute considerably to the success of genetic studies. We have shown how phenotypic measurement issues can improve the success of GWAS, and expect that phenotype-related measurement issues will attract more attention in the future [52]–[53]. Together with advances in genetic information modelling (e.g., gene-network approaches [54]–[55]), advances in phenotypic modelling can contribute substantially to the success of future gene-finding studies.

## Supporting Information

Figure S1 Factor models for two samples used to simulate configural invariance (see Table S8 for results).
(0.06 MB DOC)

Figure S2 Factor models for two samples used to simulate configural invariance (for results see Table S9).
(1.46 MB TIF)

Figure S3 Distribution of the 10.000 p-values of the regression of the sum score on a genetic variant explaining .05% of the variance for five subscales (an complete scale with 27 items, a subtest with the 9 middle items, a subtest with 9 items selected to cover the entire continuum, a subtest with 5 low-extreme and 4 high-extreme items, and a subtest with 9 high-extreme items) for the population-based design.
(1.73 MB TIF)

Figure S4 Distribution of the 10.000 p-values of the regression of the sum score on a genetic variant explaining .05% of the variance for five subscales (an complete scale with 27 items, a subtest with the 9 middle items, a subtest with 9 items selected to cover the entire continuum, a subtest with 5 low-extreme and 4 high-extreme items, and a subtest with 9 high-extreme items) for the selected samples design.
(1.37 MB TIF)

Figure S5 Test information curves for each subtest (an complete scale with 27 items, a subtest with the 9 middle items, a subtest with 9 items selected to cover the entire continuum, a subtest with 5 low-extreme and 4 high-extreme items, and a subtest with 9 high-extreme items) for the population-based design. On the x-axis of these figures are the latent trait scores, on the y-axis the information scores. Because of the assumption of local independence (i.e., conditional on the latent trait, the item scores show no additional correlation), the test information for a certain level of the latent trait is simply the sum of the information of the individual items for that level of the latent trait. The item information is calculated as: I(Θ) = a^2^_i_ * p_i_(Θ) * q_i_(Θ), where a_i_ is the discrimination parameter for the ith item (fixed to 1 in the current simulation), p_i_(Θ) is the probability of answering item i correctly for the a certain latent trait value Θ, and q_i_(Θ) is the probability of answering item i incorrectly for that latent trait value Θ. Note that the item is maximally informative for the level of the latent trait where p = q = .5, so when ai = 1, as was the case for all items in our simulation, the maximum information of an item equals .25. As the test information is the sum of all information in the individual items, the test information depends on the number of items as well as the informativeness of every individual item for a certain level of the latent trait.
(1.52 MB TIF)

Figure S6 Test information curves for each subtest (an complete scale with 27 items, a subtest with the 9 middle items, a subtest with 9 items selected to cover the entire continuum, a subtest with 5 low-extreme and 4 high-extreme items, and a subtest with 9 high-extreme items) for the selected samples design.
(1.19 MB TIF)

Table S1 2-factor model with effect genetic variant on second factor only.
(0.05 MB DOC)

Table S2 3-factor model with effect genetic variant on third factor only.
(0.05 MB DOC)

Table S3 2-factor model with effect genetic variant equally strong on both factors.
(0.05 MB DOC)

Table S4 3-factor model with effect genetic variant equally strong on all three factors.
(0.05 MB DOC)

Table S5 Unequal factors loadings 6 items.
(0.05 MB DOC)

Table S6 Unequal factors loadings 12 items.
(0.05 MB DOC)

Table S7 Unequal residual variances 6 and 12 items.
(0.05 MB DOC)

Table S8 Violations of configural invariance (equal factor structure) modeled according to Figure S1.
(0.04 MB DOC)

Table S9 Violations of configural invariance (equal factor structure) modeled according to Figure S2.
(0.04 MB DOC)

Table S10 Violations of metric invariance (equal factor loadings across samples) in the context of 6 items.
(0.05 MB DOC)

Table S11 Violations of metric invariance (equal factor loadings across samples) in the context of 12 items.
(0.04 MB DOC)

Table S12 Violations of strong factorial invariance (equal item means across samples) in the context of 6 items.
(0.04 MB DOC)

Table S13 Violations of strong factorial invariance (equal item means across samples) in the context of 12 items.
(0.04 MB DOC)

Table S14 Violations of strict factorial invariance (equal residual variances across samples) in the context of 6 items.
(0.04 MB DOC)

Table S15 Violations of strict factorial invariance (equal residual variances across samples) in the context of 12 items.
(0.04 MB DOC)

Table S16 Violation of measurement invariance with respect to the genetic variant itself.
(0.05 MB DOC)

Table S17 Results simulation study (Nsim = 10.000) into the power to detect a genetic variant explaining .6% of the variance with 5 differently constructed phenotypic instruments (an complete scale with 27 items, a subtest with the 9 middle items, a subtest with 9 items selected to cover the entire continuum, a subtest with 5 low-extreme and 4 high-extreme items, and a subtest with 9 high-extreme items) in two designs: a population design (N = 2500) and a selected-samples design (1250 extreme subjects and 1250 subjects from the normal range).
(0.05 MB DOC)

Scripts S1 Supplemental information: the scripts used for the simulations.
(0.04 MB ZIP)
