# Supplementary figures and images for: Phenotypic Complexity, Measurement Bias, and Poor Phenotypic Resolution Contribute to the Missing Heritability Problem in Genetic Association Studies
Source: PLoS One. 2010 Nov 10;5(11):e13929. doi: 10.1371/journal.pone.0013929 (PMC2978099; doi:10.1371/journal.pone.0013929)

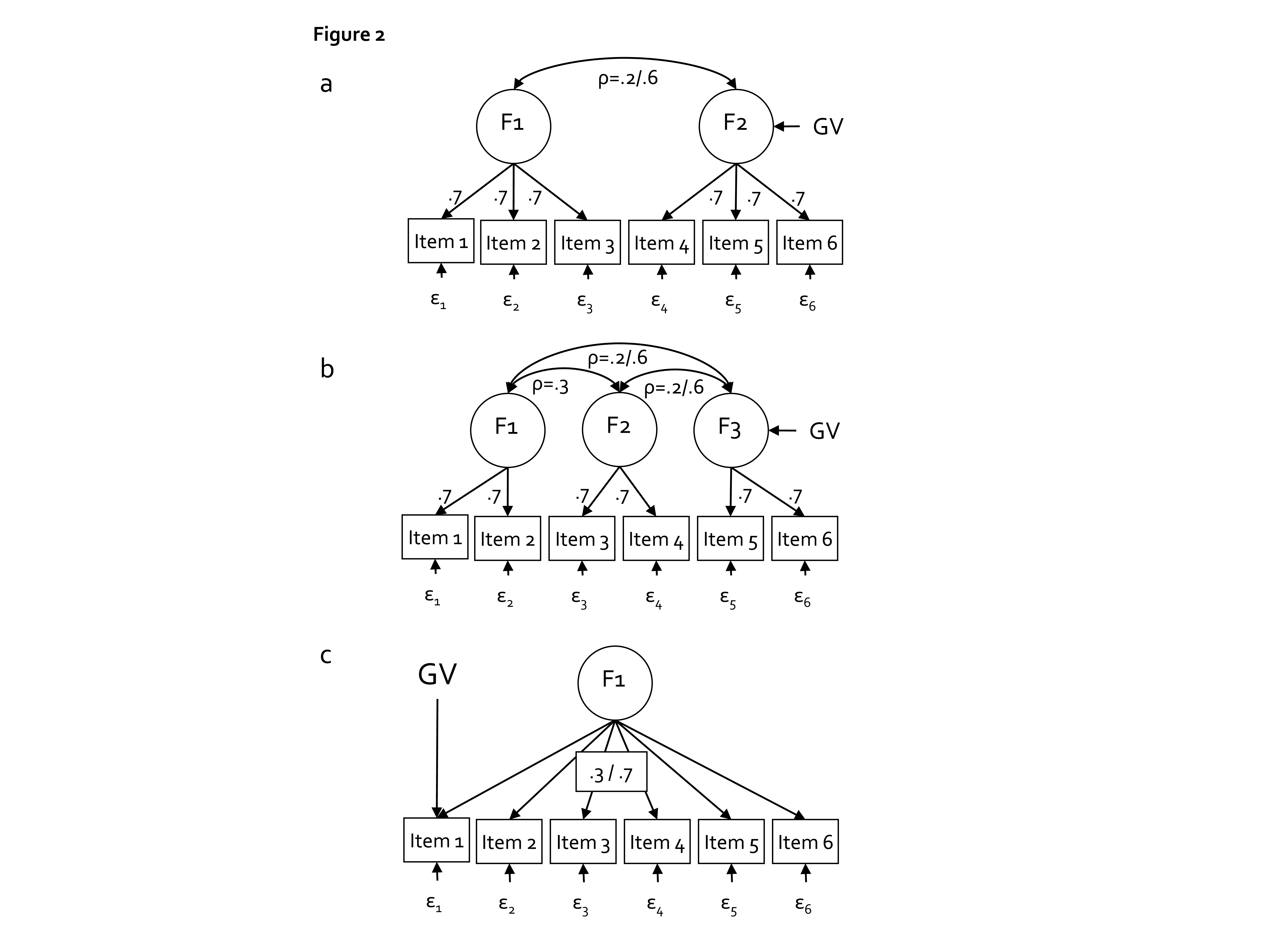

Supplement: Figure S2 — Factor models for two samples used to simulate configural invariance (for results see Table S9). (1.46 MB TIF) [file pone.0013929.s002.tif]

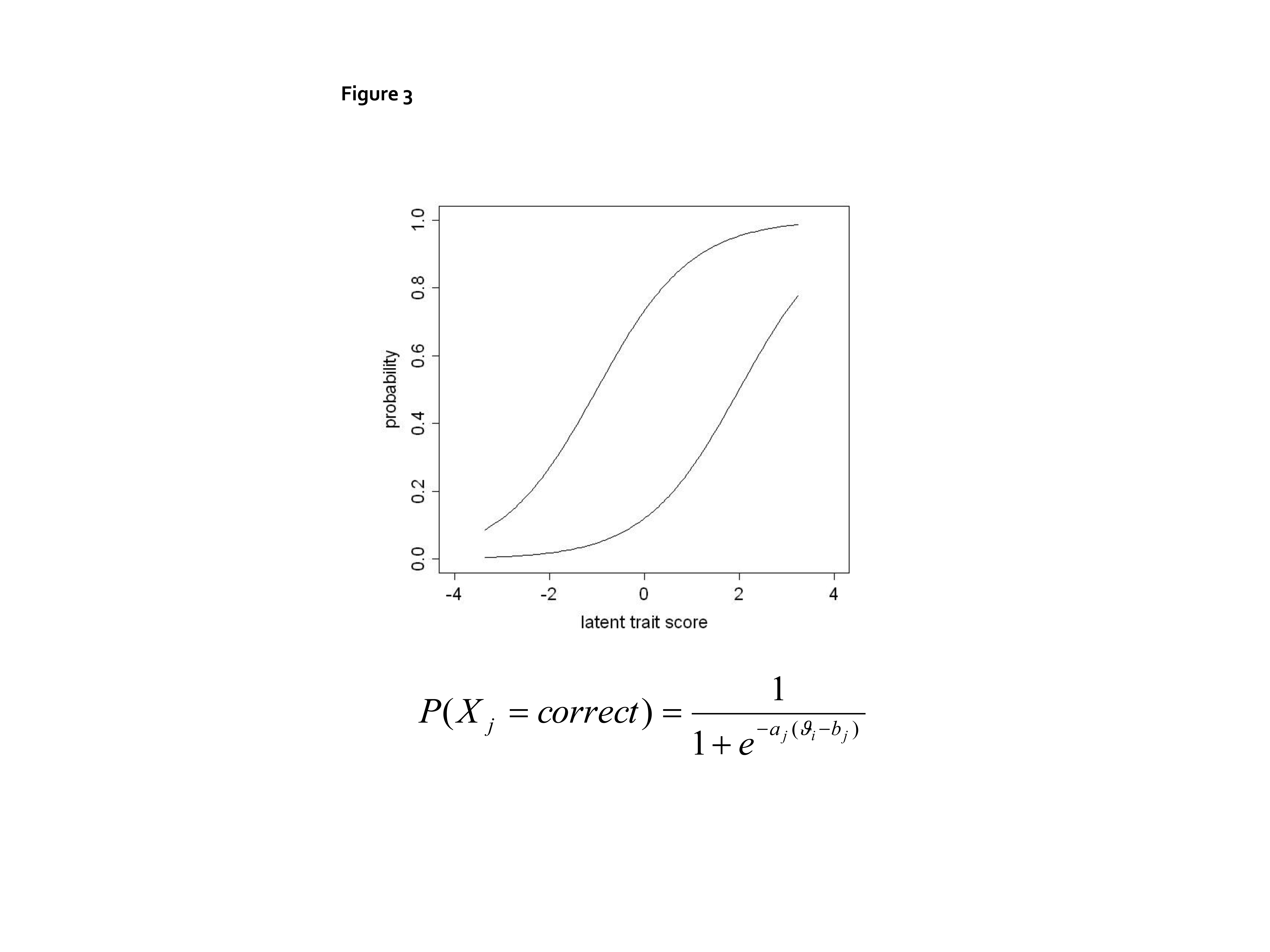

Supplement: Figure S3 — Distribution of the 10.000 p-values of the regression of the sum score on a genetic variant explaining .05% of the variance for five subscales (an complete scale with 27 items, a subtest with the 9 middle items, a subtest with 9 items selected to cover the entire continuum, a subtest with 5 low-extreme and 4 high-extreme items, and a subtest with 9 high-extreme items) for the population-based design. (1.73 MB TIF) [file pone.0013929.s003.tif]

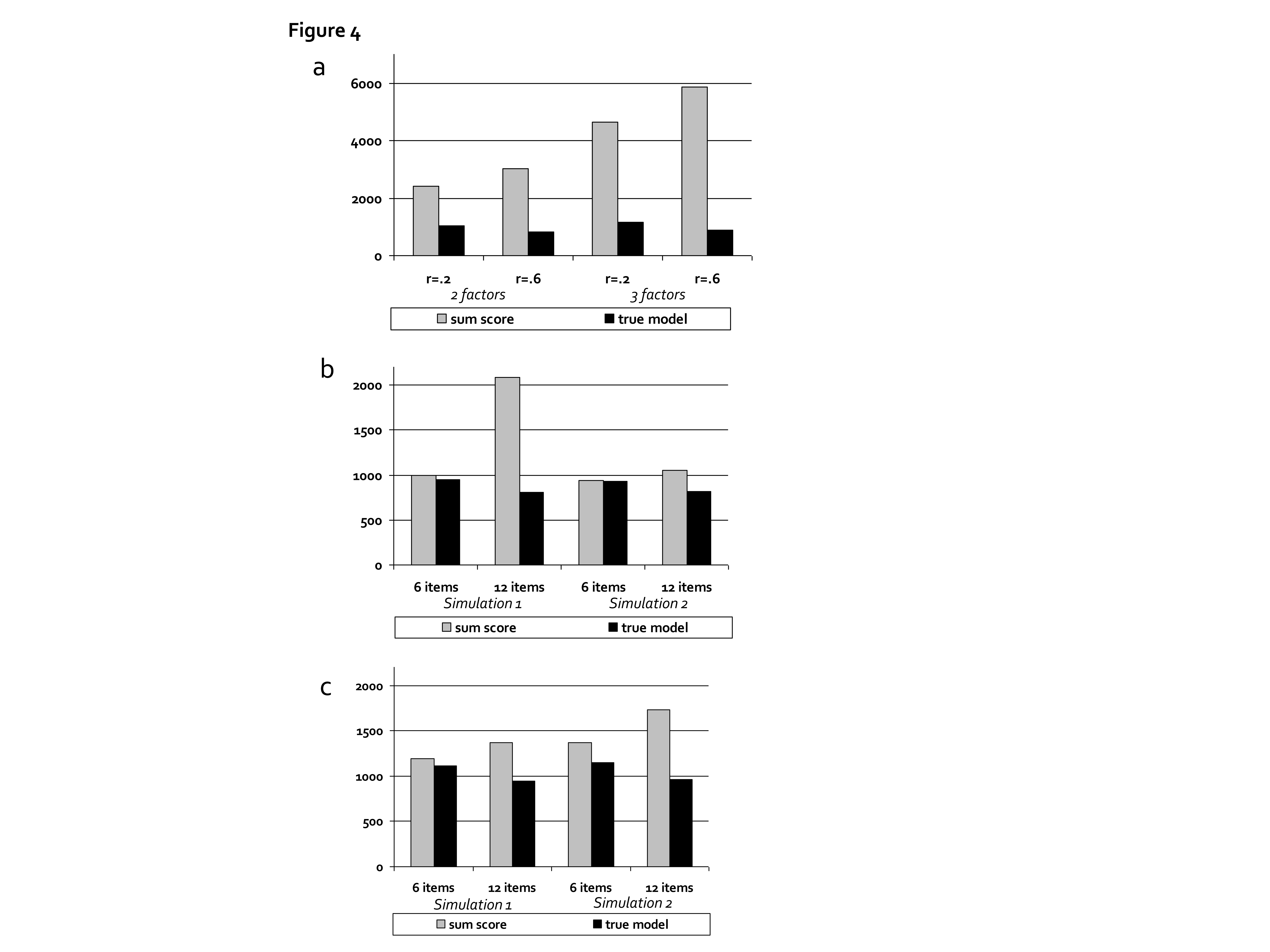

Supplement: Figure S4 — Distribution of the 10.000 p-values of the regression of the sum score on a genetic variant explaining .05% of the variance for five subscales (an complete scale with 27 items, a subtest with the 9 middle items, a subtest with 9 items selected to cover the entire continuum, a subtest with 5 low-extreme and 4 high-extreme items, and a subtest with 9 high-extreme items) for the selected samples design. (1.37 MB TIF) [file pone.0013929.s004.tif]

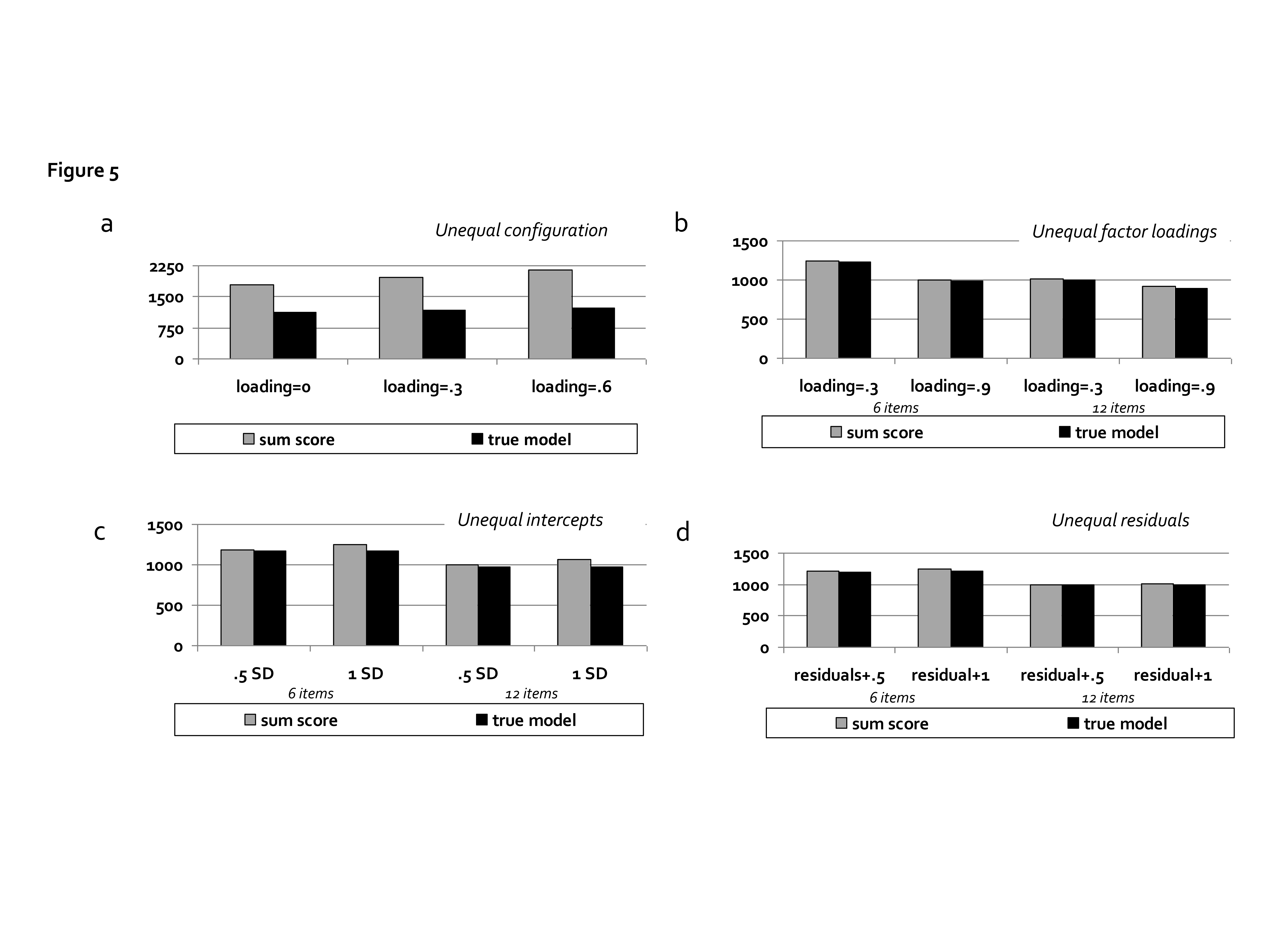

Supplement: Figure S5 — Test information curves for each subtest (an complete scale with 27 items, a subtest with the 9 middle items, a subtest with 9 items selected to cover the entire continuum, a subtest with 5 low-extreme and 4 high-extreme items, and a subtest with 9 high-extreme items) for the population-based design. On the x-axis of these figures are the latent trait scores, on the y-axis the information scores. Because of the assumption of local independence (i.e., conditional on the latent trait, the item scores show no additional correlation), the test information for a certain level of the latent trait is simply the sum of the information of the individual items for that level of the latent trait. The item information is calculated as: I(Θ) = a2 i * pi(Θ) * qi(Θ), where ai is the discrimination parameter for the ith item (fixed to 1 in the current simulation), pi(Θ) is the probability of answering item i correctly for the a certain latent trait value Θ, and qi(Θ) is the probability of answering item i incorrectly for that latent trait value Θ. Note that the item is maximally informative for the level of the latent trait where p = q = .5, so when ai = 1, as was the case for all items in our simulation, the maximum information of an item equals .25. As the test information is the sum of all information in the individual items, the test information depends on the number of items as well as the informativeness of every individual item for a certain level of the latent trait. (1.52 MB TIF) [file pone.0013929.s005.tif]

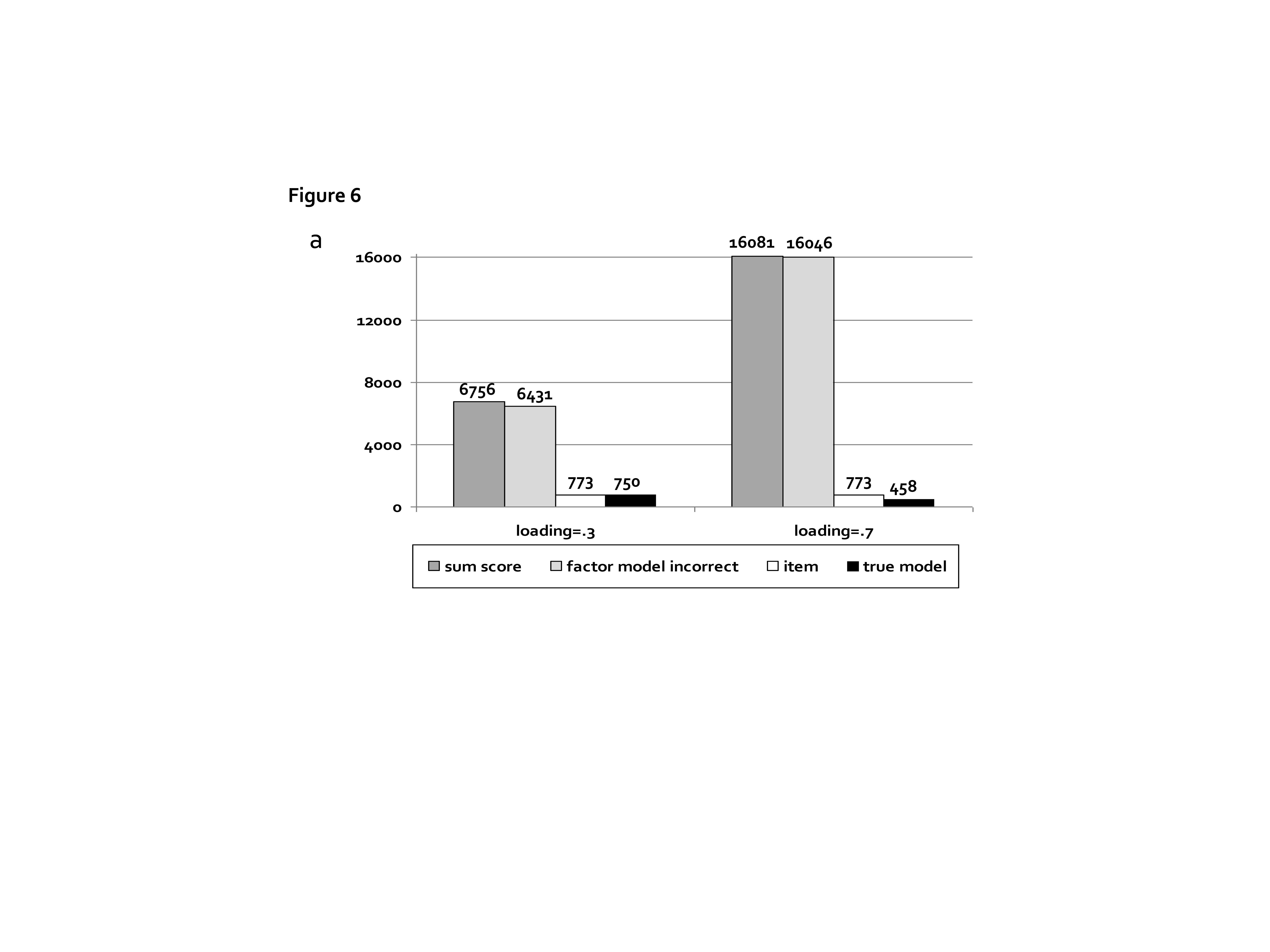

Supplement: Figure S6 — Test information curves for each subtest (an complete scale with 27 items, a subtest with the 9 middle items, a subtest with 9 items selected to cover the entire continuum, a subtest with 5 low-extreme and 4 high-extreme items, and a subtest with 9 high-extreme items) for the selected samples design. (1.19 MB TIF) [file pone.0013929.s006.tif]
