# Supplementary material for: Phenotypic Complexity, Measurement Bias, and Poor Phenotypic Resolution Contribute to the Missing Heritability Problem in Genetic Association Studies
Source: PLoS One. 2010 Nov 10;5(11):e13929. doi: 10.1371/journal.pone.0013929 (PMC2978099; doi:10.1371/journal.pone.0013929)
Supplement: Table S1 — 2-factor model with effect genetic variant on second factor only. (0.05 MB DOC) [file pone.0013929.s007.doc]

**Supplemental Data**

**Supplement to**

“Phenotypic complexity, measurement bias, and poor phenotypic resolution contribute to the missing heritability problem in genetic association studies”

Sophie van der Sluis

Matthijs Verhage

Danielle Posthuma

Conor V. Dolan

| Table S1: violations unidimensionality. 2-factor model rather than 1-factor model with genetic variant explaining 1% of the variance in the second factor only | | | | | | |
| --- | --- | --- | --- | --- | --- | --- |
|  |  |  |  |  |  |  |
|  | **cor[2,1]=.2** | | | **cor[2,1]=.6** | | |
|  | **χ^2^(1)** | **Observed**  **Power for N=1200** | **N required for**  **power of .80** | **χ^2^(1)** | **Observed power for N=1200** | **N required for**  **power of .80** |
| **P=.5** |  |  |  |  |  |  |
| Sum | 3.916 | .51 | 2405 | 3.112 | .42 | 3027 |
| True | 9.190 | .86 | 1025 | 11.209 | .912 | 840 |
| **P=.3** |  |  |  |  |  |  |
| Sum | 3.289 | .44 | 2863 | 2.614 | .37 | 3603 |
| True | 7.721 | .79 | 1220 | 9.481 | .87 | 100 |
|  |  |  |  |  |  |  |
| Note: Data were simulated according to a 2 factor model, with the genetic variants explaining 1% of the variance in the second factor only. cor[2,1] denotes the correlation between the first and second factor. P denotes the frequency of the first allele of the diallelic GV. χ^2^(1) denotes the increase in likelihood when the regression between the GV and the trait is fixed to 0 (a 1-df test). N denotes the sample size required for a power of 80% when α=.05. | | | | | | |
