# Supplementary material for: Phenotypic Complexity, Measurement Bias, and Poor Phenotypic Resolution Contribute to the Missing Heritability Problem in Genetic Association Studies
Source: PLoS One. 2010 Nov 10;5(11):e13929. doi: 10.1371/journal.pone.0013929 (PMC2978099; doi:10.1371/journal.pone.0013929)
Supplement: Table S2 — 3-factor model with effect genetic variant on third factor only. (0.05 MB DOC) [file pone.0013929.s008.doc]

**Supplemental Data**

**Supplement to**

“Phenotypic complexity, measurement bias, and poor phenotypic resolution contribute to the missing heritability problem in genetic association studies”

Sophie van der Sluis

Matthijs Verhage

Danielle Posthuma

Conor V. Dolan

| Table S2: violations unidimensionality. 3-factor model rather than 1-factor model with genetic variant explaining 1% of the variance in the third factor only | | | | | | |
| --- | --- | --- | --- | --- | --- | --- |
|  |  |  |  |  |  |  |
|  | **cor[3,1/2]=.2** | | | **cor[3,1/2]=.6** | | |
|  | **χ^2^(1)** | **Observed power for N=1200** | **N required for**  **power of .80** | **χ^2^(1)** | **Observed power for N=1200** | **N required for**  **power of .80** |
| **P=.5** |  |  |  |  |  |  |
| Sum | 2.033 | .297 | 4632 | 1.603 | .24 | 5875 |
| True | 8.210 | .817 | 1147 | 10.776 | .91 | 874 |
| **P=.3** |  |  |  |  |  |  |
| Sum | 1.708 | .26 | 5514 | 1.347 | .21 | 6994 |
| True | 6.896 | .75 | 1366 | 9.051 | .85 | 1041 |
|  |  |  |  |  |  |  |
| Note: Data were simulated according to a 3 factor model, with the genetic variants explaining 1% of the variance in the third factor only. cor[3,1/2] denotes the correlation between the third factor, and the first and second factor. The correlation between the first two factors was fixed to .3. P denotes the frequency of the first allele of the diallelic GV. χ^2^(1) denotes the increase in likelihood when the regression between the GV and the trait is fixed to 0 (a 1-df test). N denotes the sample size required for a power of 80% when α=.05. | | | | | | |
