# Supplementary material for: Phenotypic Complexity, Measurement Bias, and Poor Phenotypic Resolution Contribute to the Missing Heritability Problem in Genetic Association Studies
Source: PLoS One. 2010 Nov 10;5(11):e13929. doi: 10.1371/journal.pone.0013929 (PMC2978099; doi:10.1371/journal.pone.0013929)
Supplement: Table S3 — 2-factor model with effect genetic variant equally strong on both factors. (0.05 MB DOC) [file pone.0013929.s009.doc]

**Supplemental Data**

**Supplement to**

“Phenotypic complexity, measurement bias, and poor phenotypic resolution contribute to the missing heritability problem in genetic association studies”

Sophie van der Sluis

Matthijs Verhage

Danielle Posthuma

Conor V. Dolan

| Table S3: violations of unidimensionality. 2 factor model with a genetic variant affecting both factors equally | | | | | | |
| --- | --- | --- | --- | --- | --- | --- |
|  |  |  |  |  |  |  |
|  | **cor[2,1]=.2** | | | **cor[2,1]=.6** | | |
|  | **χ^2^(1)** | **Obs power N=1200** | **N required for power of 80%** | **χ^2^(1)** | **Obs power N=1200** | **N required for power of 80%** |
| **P=.5** |  |  |  |  |  |  |
| Sum | 15.587 | .98 | 604 | 12.398 | .94 | 760 |
| true | 15.587 | .98 | 604 | 12.398 | .94 | 760 |
| **P=.3** |  |  |  |  |  |  |
| Sum | 13.104 | .95 | 719 | 10.422 | .90 | 904 |
| true | 13.104 | .95 | 719 | 10.422 | .90 | 904 |
|  |  |  |  |  |  |  |
| Note: cor[2,1] denotes the correlation between the second factor, and the first factor. P denotes the frequency of the first allele of the diallelic GV. χ^2^(1) denotes the increase in likelihood when the regression between the GV and the trait is fixed to 0 (a 1-df test). N denotes the sample size required for a power of 80% when α=.05. | | | | | | |
