# Supplementary material for: Phenotypic Complexity, Measurement Bias, and Poor Phenotypic Resolution Contribute to the Missing Heritability Problem in Genetic Association Studies
Source: PLoS One. 2010 Nov 10;5(11):e13929. doi: 10.1371/journal.pone.0013929 (PMC2978099; doi:10.1371/journal.pone.0013929)
Supplement: Table S5 — Unequal factors loadings 6 items. (0.05 MB DOC) [file pone.0013929.s011.doc]

**Supplemental Data**

**Supplement to**

“Phenotypic complexity, measurement bias, and poor phenotypic resolution contribute to the missing heritability problem in genetic association studies”

Sophie van der Sluis

Matthijs Verhage

Danielle Posthuma

Conor V. Dolan

| Table S5: violations equal factor loadings (6 items) | | | | | | | | |
| --- | --- | --- | --- | --- | --- | --- | --- | --- |
|  |  |  |  |  |  |  |  |  |
|  | **L=.3.3.3 .5.5.5** | | **L=.3.3.3 .9.9.9** | | **L=.3.3.5.5.7.7** | | **L=.5.5.7.7.9.9** | |
|  | **χ^2^** | **N required for**  **power of .80 (observed power)** | **χ^2^** | **N required for**  **power of .80 (observed power)** | **χ^2^** | **N required for**  **power of .80 (observed power)** | **χ^2^** | **N required for**  **power of .80 (observed power)** |
| **P=.5** |  |  |  |  |  |  |  |  |
| Sum | 7.436 | 1266 (.78) | 9.449 | 997 (.87) | 8.627 | 1092 (.84) | 10.025 | 939 (.89) |
| True | 7.608 | 1238 (.79) | 9.877 | 954 (.88) | 8.870 | 1062 (.85) | 10.113 | 931 (.89) |
| **P=.3** |  |  |  |  |  |  |  |  |
| Sum | 6.249 | 1507 (.71) | 7.942 | 1186 (.80) | 7.251 | 1299 (.77) | 8.426 | 1117 (.83) |
| True | 6.394 | 1473 (.72) | 8.302 | 1135 (.82) | 7.455 | 1263 (.78) | 8.501 | 1108 (.83) |
|  |  |  |  |  |  |  |  |  |
| Note: L denotes the pattern of factor loadings for the 6 items on the one latent factor. P denotes the frequency of the first allele of the diallelic GV. χ^2^(1) denotes the increase in likelihood when the regression between the GV and the trait is fixed to 0 (a 1-df test). N denotes the sample size required for a power of 80% when α=.05. Between brackets, the observed power for N=1200 is shown. | | | | | | | | |
