# Supplementary material for: Phenotypic Complexity, Measurement Bias, and Poor Phenotypic Resolution Contribute to the Missing Heritability Problem in Genetic Association Studies
Source: PLoS One. 2010 Nov 10;5(11):e13929. doi: 10.1371/journal.pone.0013929 (PMC2978099; doi:10.1371/journal.pone.0013929)
Supplement: Table S6 — Unequal factors loadings 12 items. (0.05 MB DOC) [file pone.0013929.s012.doc]

**Supplemental Data**

**Supplement to**

“Phenotypic complexity, measurement bias, and poor phenotypic resolution contribute to the missing heritability problem in genetic association studies”

Sophie van der Sluis

Matthijs Verhage

Danielle Posthuma

Conor V. Dolan

| Table S6: violations equal factor loadings (12 items) | | | | | | |
| --- | --- | --- | --- | --- | --- | --- |
|  |  |  |  |  |  |  |
|  | **L=.3.3.3.3.3.3 .9.9.9.9.9.9** | | | **L=.5.5.5.5.7.7.7.7.9.9** | | |
|  | **χ^2^** | **Observed power given N=1200** | **N required for**  **power of .80** | **χ^2^** | **Observed power given N=1200** | **N required for**  **power of .80** |
| **P=.5** |  |  |  |  |  |  |
| Sum | 4.508 | .56 | 2089 | 8.972 | .85 | 1050 |
| Factor | 11.636 | .93 | 809 | 11.561 | .93 | 815 |
| **P=.3** |  |  |  |  |  |  |
| Sum | 3.788 | .49 | 2487 | 7.539 | .78 | 1249 |
| Factor | 9.780 | .88 | 963 | 9.716 | .88 | 969 |
|  |  |  |  |  |  |  |
| Note: L denotes the pattern of factor loadings for the 12 items on the one latent factor. P denotes the frequency of the first allele of the diallelic GV. χ^2^(1) denotes the increase in likelihood when the regression between the GV and the trait is fixed to 0 (a 1-df test). N denotes the sample size required for a power of 80% when α=.05. | | | | | | |
