# Supplementary material for: Phenotypic Complexity, Measurement Bias, and Poor Phenotypic Resolution Contribute to the Missing Heritability Problem in Genetic Association Studies
Source: PLoS One. 2010 Nov 10;5(11):e13929. doi: 10.1371/journal.pone.0013929 (PMC2978099; doi:10.1371/journal.pone.0013929)
Supplement: Table S7 — Unequal residual variances 6 and 12 items. (0.05 MB DOC) [file pone.0013929.s013.doc]

**Supplemental Data**

**Supplement to**

“Phenotypic complexity, measurement bias, and poor phenotypic resolution contribute to the missing heritability problem in genetic association studies”

Sophie van der Sluis

Matthijs Verhage

Danielle Posthuma

Conor V. Dolan

| Table S7: violation equal residual variances | | | | | | | | | | | | | |
| --- | --- | --- | --- | --- | --- | --- | --- | --- | --- | --- | --- | --- | --- |
|  |  |  |  |  |  |  |  |  |  |  |  |  |  |
|  | Nitems=6 | | | | | |  | Nitems=12 | | | | | |
|  |  |  |  |  |  |  |  |  |  |  |  |  |  |
|  | **ε=.64,.64,.64,**  **1.14,1.14,.1.14** | | **ε=.64,.64,.64,**  **1.64,1.64,1.64** | | **ε=.64,.64,.64,**  **2.64,2.64,2.64** | |  | **ε=.64,.64,.64,**  **.64,.64,.64**  **1.14,1.14,.1.14, 1.14,1.14,.1.14** | | **ε=.64,.64,.64,**  **.64,.64,.64**  **1.64,1.64,1.64**  **1.64,1.64,1.64** | | **ε=.64,.64,.64,**  **.64,.64,.64**  **2.64,2.64,2.64**  **2.64,2.64,2.64** | |
|  | **χ^2^(1)** | **N** | **χ^2^(1)** | **N** | **χ^2^(1)** | **N** |  | **χ^2^(1)** | **N** | **χ^2^(1)** | **N** | **χ^2^(1)** | **N** |
| **P=.5** |  |  |  |  |  |  |  |  |  |  |  |  |  |
| Sum | 8.564 | 1099 (.83) | 7.916 | 1189 (.80) | 6.877 | 1369 (.75) |  | 7.917 | 1189 (.80) | 6.877 | 1369 (.75) | 5.447 | 1729 (.65) |
| 1-factor | 8.766 | 1074 (.84) | 8.481 | 1110 (.83) | 8.192 | 1149 (.82) |  | 10.152 | 927 (.89) | 9.959 | 945 (.88) | 9.756 | 965 (.88) |
| **P=.3** |  |  |  |  |  |  |  |  |  |  |  |  |  |
| Sum | 7.196 | 1309 (.76) | 6.652 | 1416 (.73) | 5.778 | 1630 (.67) |  | 6.652 | 1416 (.73) | 5.779 | 1630 (.67) | 4.576 | 2058 (.57) |
| 1-factor | 7.366 | 1279 (.77) | 7.126 | 1322 (.76) | 6.883 | 1368 (.75) |  | 8.533 | 1104 (.83) | 8.37 | 1125 (.82) | 8.200 | 1149 (.82) |
|  |  |  |  |  |  |  |  |  |  |  |  |  |  |
| Note: ε denotes the pattern of residual variances for the 6 or 12 items. P denotes the frequency of the first allele of the diallelic GV. χ^2^(1) denotes the increase in likelihood when the regression between the GV and the trait is fixed to 0 (a 1-df test). N denotes the sample size required for a power of 80% when α=.05. The observed power for N=1200 is shown between brackets. | | | | | | | | | | | | | |
