# Supplementary material for: Phenotypic Complexity, Measurement Bias, and Poor Phenotypic Resolution Contribute to the Missing Heritability Problem in Genetic Association Studies
Source: PLoS One. 2010 Nov 10;5(11):e13929. doi: 10.1371/journal.pone.0013929 (PMC2978099; doi:10.1371/journal.pone.0013929)
Supplement: Table S8 — Violations of configural invariance (equal factor structure) modeled according to Figure S1. (0.04 MB DOC) [file pone.0013929.s014.doc]

**Supplemental Data**

**Supplement to**

“Phenotypic complexity, measurement bias, and poor phenotypic resolution contribute to the missing heritability problem in genetic association studies”

Sophie van der Sluis

Matthijs Verhage

Danielle Posthuma

Conor V. Dolan

| **Table S8: Configural invariance modelled as shown in Figures 1a/b of this Supplement** | | | | | | |
| --- | --- | --- | --- | --- | --- | --- |
|  |  | | | | | |
|  | **Corr (F1,F2)=.3** | | | | | |
|  | **L=0** |  | **L=.3** |  | **L=.6** |  |
|  | **χ^2^** | **N** | **χ^2^** | **N** | **χ^2^** | **N** |
| **P=.5** |  |  |  |  |  |  |
| Sum | 5.248 | 1795 (.63) | 4.806 | 1960 (.59) | 4.374 | 2153 (.55) |
| 2 group factor (true) | 8.306 | 1134 (.82) | 7.934 | 1187 (.80) | 7.629 | 1235 (.79) |
|  |  |  |  |  |  |  |
| Note: Corr(F1,F2) denotes the correlation between the first factor (not affected by the GV) and the second factor (affected by the GV). P denotes the frequency of the first allele of the diallelic GV. χ^2^(1) denotes the increase in likelihood when the regression between the GV and the trait is fixed to 0 (a 1-df test). N denotes the sample size required for a power of 80% when α=.05. The observed power for N=1200 is shown between brackets. | | | | | | |
