# Supplementary material for: Phenotypic Complexity, Measurement Bias, and Poor Phenotypic Resolution Contribute to the Missing Heritability Problem in Genetic Association Studies
Source: PLoS One. 2010 Nov 10;5(11):e13929. doi: 10.1371/journal.pone.0013929 (PMC2978099; doi:10.1371/journal.pone.0013929)
Supplement: Table S11 — Violations of metric invariance (equal factor loadings across samples) in the context of 12 items. (0.04 MB DOC) [file pone.0013929.s017.doc]

**Supplemental Data**

**Supplement to**

“Phenotypic complexity, measurement bias, and poor phenotypic resolution contribute to the missing heritability problem in genetic association studies”

Sophie van der Sluis

Matthijs Verhage

Danielle Posthuma

Conor V. Dolan

| Table S11. violations of metric invariance in the context of 12 items | | | | | | | | | | | | |
| --- | --- | --- | --- | --- | --- | --- | --- | --- | --- | --- | --- | --- |
|  |  |  |  |  |  |  |  |  |  |  |  |  |
|  | **L1=.5.5.5.5.5.5**  **L1=.3.3.5.5.5.5** | | | **L1=.5.5.5.5.5.5**  **L1=.5.5.5.5.5.5** | | | **L1=.5.5.5.5.5.5**  **L1=.7.7.5.5.5.5** | | | **L1=.5.5.5.5.5.5**  **L1=.9.9.5.5.5.5** | | |
|  | **χ^2^** | **pow** | **N** | **χ^2^** | **pow** | **N** | **χ^2^** | **pow** | **N** | **χ^2^** | **pow** | **N** |
| **P=.5** |  |  |  |  |  |  |  |  |  |  |  |  |
| Sum | 9.351 | .86 | 1007 | 9.665 | .87 | 975 | 9.871 | .88 | 954 | 9.993 | .89 | 943 |
| 2gr factor true | 9.417 | .87 | 1000 | 9.665 | .87 | 975 | 9.909 | .88 | 951 | 10.113 | .89 | 931 |
|  |  |  |  |  |  |  |  |  |  |  |  |  |
| Note: L1 and L2 denote the vectors of factor loadings for items 1 to 12 in samples 1 and 2 respectively. P denotes the frequencies of the first allele of the diallelic GV. χ^2^(1) denotes the increase in likelihood when the regression between the GV and the trait is fixed to 0 (a 1-df test). N denotes the sample size required for a power of 80% when α=.05. Between brackets, the observed power for N=1200 is shown. | | | | | | | | | | | | |
