# Supplementary material for: Phenotypic Complexity, Measurement Bias, and Poor Phenotypic Resolution Contribute to the Missing Heritability Problem in Genetic Association Studies
Source: PLoS One. 2010 Nov 10;5(11):e13929. doi: 10.1371/journal.pone.0013929 (PMC2978099; doi:10.1371/journal.pone.0013929)
Supplement: Table S12 — Violations of strong factorial invariance (equal item means across samples) in the context of 6 items. (0.04 MB DOC) [file pone.0013929.s018.doc]

**Supplemental Data**

**Supplement to**

“Phenotypic complexity, measurement bias, and poor phenotypic resolution contribute to the missing heritability problem in genetic association studies”

Sophie van der Sluis

Matthijs Verhage

Danielle Posthuma

Conor V. Dolan

| Table S12: Violations of strong factorial invariance in the context of 6 items | | | | | | | | |
| --- | --- | --- | --- | --- | --- | --- | --- | --- |
|  |  |  |  |  |  |  |  |  |
|  | **M1=000000**  **M2=000000** |  | **M1=000000**  **M2=.1.10000** |  | **M1=000000**  **M2=.5.50000** |  | **M1=000000**  **M2=110000** |  |
|  | **χ^2^** | **N** | **χ^2^** | **N** | **χ^2^** | **N** | **χ^2^** | **N** |
| **P=.5** |  |  |  |  |  |  |  |  |
| Sum | 8.063 | 1168 (.81) | 8.057 | 1169 (.81) | 7.916 | 1190 (.80) | 7.508 | 1255 (.78) |
| 2gr factor true | 8.063 | 1168 (.81) | 8.063 | 1168 (.81) | 8.063 | 1168 (.81) | 8.063 | 1168 (.81) |
|  |  |  |  |  |  |  |  |  |
| Note: M1 and M2 denote the vectors of observed means for items 1 to 6 in samples 1 and 2 respectively. P denotes the frequencies of the first allele of the diallelic GV. χ^2^(1) denotes the increase in likelihood when the regression between the GV and the trait is fixed to 0 (a 1-df test). N denotes the sample size required for a power of 80% when α=.05. Between brackets, the observed power for N=1200 is shown. | | | | | | | | |
