# Supplementary material for: Phenotypic Complexity, Measurement Bias, and Poor Phenotypic Resolution Contribute to the Missing Heritability Problem in Genetic Association Studies
Source: PLoS One. 2010 Nov 10;5(11):e13929. doi: 10.1371/journal.pone.0013929 (PMC2978099; doi:10.1371/journal.pone.0013929)
Supplement: Table S15 — Violations of strict factorial invariance (equal residual variances across samples) in the context of 12 items. (0.04 MB DOC) [file pone.0013929.s021.doc]

**Supplemental Data Description**

**Supplement to**

“Phenotypic complexity, measurement bias, and poor phenotypic resolution contribute to the missing heritability problem in genetic association studies”

Sophie van der Sluis

Matthijs Verhage

Danielle Posthuma

Conor V. Dolan

| Table S15: Violations of strict factorial invariance in the context of 12 items | | | | | | | | |
| --- | --- | --- | --- | --- | --- | --- | --- | --- |
|  |  |  |  |  |  |  |  |  |
|  | **e1=.747 for all**  **e2=.747 for all** | | **e1=.747 for all**  **e2=.747+.5 for the first 4 items** | | **e1=.747 for all**  **e2=.747+1 for the first 4 items** | | **e1=.747 for all**  **e2=.747+2 for the first 4 items** | |
|  | **χ^2^** | **N** | **χ^2^** | **N** | **χ^2^** | **N** | **χ^2^** | **N** |
| **P=.5** |  |  |  |  |  |  |  |  |
| Sum | 9.665 | 975 (.99) | 9.455 | 996 (.99) | 9.255 | 1018 (.99) | 8.878 | 1061 (.99) |
| Eta-2gr 6item | 9.665 | 975 (.99) | 9.521 | 989 (.99) | 9.449 | 997 (.99) | 9.376 | 1005 (.99) |
|  |  |  |  |  |  |  |  |  |
| Note: e1 and e2 denote the vectors of residual variances for items 1 to 12 in samples 1 and 2 respectively. P denotes the frequencies of the first allele of the diallelic GV. χ^2^(1) denotes the increase in likelihood when the regression between the GV and the trait is fixed to 0 (a 1-df test). N denotes the sample size required for a power of 80% when α=.05. Between brackets, the observed power for N=1200 is shown. | | | | | | | | |
