# Supplementary material for: Phenotypic Complexity, Measurement Bias, and Poor Phenotypic Resolution Contribute to the Missing Heritability Problem in Genetic Association Studies
Source: PLoS One. 2010 Nov 10;5(11):e13929. doi: 10.1371/journal.pone.0013929 (PMC2978099; doi:10.1371/journal.pone.0013929)
Supplement: Table S16 — Violation of measurement invariance with respect to the genetic variant itself. (0.05 MB DOC) [file pone.0013929.s022.doc]

**Supplemental Data**

**Supplement to**

“Phenotypic complexity, measurement bias, and poor phenotypic resolution contribute to the missing heritability problem in genetic association studies”

Sophie van der Sluis

Matthijs Verhage

Danielle Posthuma

Conor V. Dolan

| Table S16: Violation of measurement invariance with respect to the genetic variant itself | | | | | | |
| --- | --- | --- | --- | --- | --- | --- |
|  |  |  |  |  |  |  |
|  | **L=.3** |  |  | **L=.7** |  |  |
|  | **χ^2^** | **N** |  | **χ^2^** | **N** |  |
| **P=.5** |  |  |  |  |  |  |
| Variable | 12.183 | 773 (1) |  | 12.183 | 773 (1) |  |
| Sum | 1.396 | 6756 (.22) |  | .586 | 16081 (.12) |  |
| 1-factor incorrect | 1.465 | 6431 (.23) |  | .587 | 16046 (.12) |  |
| 1-factor correct | 12.559 | 750 (1) |  | 20.567 | 458 (1) |  |
|  |  |  |  |  |  |  |
| Note: L denotes the factor loadings of all 6 items. P denotes the frequencies of the first allele of the diallelic GV. χ^2^(1) denotes the increase in likelihood when the regression between the GV and the trait is fixed to 0 (a 1-df test). N denotes the sample size required for a power of 80% when α=.05. Between brackets, the observed power for N=1200 is shown. | | | | | | |
