# Supplementary material for: Phenotypic Complexity, Measurement Bias, and Poor Phenotypic Resolution Contribute to the Missing Heritability Problem in Genetic Association Studies
Source: PLoS One. 2010 Nov 10;5(11):e13929. doi: 10.1371/journal.pone.0013929 (PMC2978099; doi:10.1371/journal.pone.0013929)
Supplement: Table S17 — Results simulation study (Nsim = 10.000) into the power to detect a genetic variant explaining .6% of the variance with 5 differently constructed phenotypic instruments (an complete scale with 27 items, a subtest with the 9 middle items, a subtest with 9 items selected to cover the entire continuum, a subtest with 5 low-extreme and 4 high-extreme items, and a subtest with 9 high-extreme items) in two designs: a population design (N = 2500) and a selected-samples design (1250 extreme subjects and 1250 subjects from the normal range). (0.05 MB DOC) [file pone.0013929.s023.doc]

**Supplemental Data**

**Supplement to**

“Phenotypic complexity, measurement bias, and poor phenotypic resolution contribute to the missing heritability problem in genetic association studies”

Sophie van der Sluis

Matthijs Verhage

Danielle Posthuma

Conor V. Dolan

| Table S17. Results simulation study (Nsim=10.000) into the power to detect a genetic variant explaining **.6%** of the variance with 5 differently constructed phenotypic instruments (an complete scale with 27 items, a subtest with the 9 middle items, a subtest with 9 items selected to cover the entire continuum, a subtest with 5 low-extreme and 4 high-extreme items, and a subtest with 9 high-extreme items) in two designs: a population design (N=2500) and a selected-samples design (1250 extreme subjects and 1250 subjects from the normal range) | | | | | | | | | | | | |
| --- | --- | --- | --- | --- | --- | --- | --- | --- | --- | --- | --- | --- |
|  |  |  |  |  |  |  |  |  |  |  |  |  |
|  | α=.05 | | | | α=.01 | | | | α=.001 | | | |
|  | population | | Selected samples | | population | | Selected samples | | population | | Selected samples | |
|  | #hits | ratio | #hits | Ratio | #hits | ratio | #hits | Ratio | #hits | ratio | #hits | ratio |
| All 27 items | 9735 |  | 9992 |  | 9075 |  | 9923 |  | 7329 |  | 9607 |  |
| 9 middle items | 9514 | .98 | 9965 | 1.00 | 8534 | .94 | 9809 | .99 | 6307 | .86 | 9195 | .96 |
| 9 high extreme | 7427 | .76 | 9920 | .99 | 5160 | .57 | 9632 | .97 | 2544 | .35 | 8570 | .89 |
| 9 items across the scale | 8815 | .91 | 9927 | .99 | 7106 | .78 | 9660 | .97 | 4385 | .60 | 8553 | .89 |
| 5 low-extreme + 4 high-extreme | 4362 | .45 | 8785 | .88 | 2177 | .24 | 7017 | .71 | 679 | .09 | 4266 | .44 |
|  |  |  |  |  |  |  |  |  |  |  |  |  |
|  |  |  |  |  |  |  |  |  |  |  |  |  |
| Note. #hits denotes the number of p-values < α=.05, α=.01, or α=.001, respectively. Ratio denotes the % of hits that the 4 subscales pick up, compared to the full instrument including all 27 items. | | | | | | | | | | | | |
